# Exploring the diversity of cancer-associated fibroblasts: insights into mechanisms of drug resistance

**DOI:** 10.3389/fcell.2024.1403122

**Published:** 2024-05-16

**Authors:** Anastasia N. Kazakova, Maria M. Lukina, Ksenia S. Anufrieva, Irina V. Bekbaeva, Olga M. Ivanova, Polina V. Shnaider, Andrey Slonov, Georgij P. Arapidi, Victoria O. Shender

**Affiliations:** ^1^ Lopukhin Federal Research and Clinical Center of Physical-Chemical Medicine of Federal Medical Biological Agency, Moscow, Russia; ^2^ Moscow Institute of Physics and Technology (National Research University), Dolgoprudny, Russia; ^3^ Center for Precision Genome Editing and Genetic Technologies for Biomedicine, Lopukhin Federal Research and Clinical Center of Physical-Chemical Medicine of Federal Medical Biological Agency, Moscow, Russia; ^4^ Institute of Experimental Oncology and Biomedical Technologies, Privolzhsky Research Medical University, Nizhny Novgorod, Russia; ^5^ Institute for Regenerative Medicine, Sechenov University, Moscow, Russia; ^6^ Faculty of Biology, Lomonosov Moscow State University, Moscow, Russia; ^7^ Shemyakin–Ovchinnikov Institute of Bioorganic Chemistry of the Russian Academy of Sciences, Moscow, Russia

**Keywords:** cancer-associated fibroblast heterogeneity, cancer-associated fibroblast populations, biomarkers, single-cell transcriptomics, tumor microenvironment, cancer disease

## Abstract

**Introduction:** Among the various stromal cell types within the tumor microenvironment, cancer-associated fibroblasts (CAFs) emerge as the predominant constituent, exhibiting a diverse array of oncogenic functions not intrinsic to normal fibroblasts. Their involvement spans across all stages of tumorigenesis, encompassing initiation, progression, and metastasis. Current understanding posits the coexistence of distinct subpopulations of CAFs within the tumor microenvironment across a spectrum of solid tumors, showcasing both pro- and antitumor activities. Recent advancements in single-cell transcriptomics have revolutionized our ability to meticulously dissect the heterogeneity inherent to CAF populations. Furthermore, accumulating evidence underscores the pivotal role of CAFs in conferring therapeutic resistance to tumors against various drug modalities. Consequently, efforts are underway to develop pharmacological agents specifically targeting CAFs.

**Methods:** This review embarks on a comprehensive analysis, consolidating data from 36 independent single-cell RNA sequencing investigations spanning 17 distinct human malignant tumor types.

**Results:** Our exploration centers on elucidating CAF population markers, discerning their prognostic relevance, delineating their functional contributions, and elucidating the underlying mechanisms orchestrating chemoresistance.

**Discussion:** Finally, we deliberate on the therapeutic potential of harnessing CAFs as promising targets for intervention strategies in clinical oncology.

## Introduction

Cancer-associated fibroblasts (CAFs) constitute a pivotal cellular component within the tumor microenvironment (TME), exerting multifaceted regulatory roles in tumorigenesis and disease progression (Ping et al., 2021). Diverging from their quiescent counterparts, CAFs are predominantly localized within the stromal compartment surrounding tumors, characterized by distinct phenotypic and functional attributes. These aberrant fibroblasts actively participate in extracellular matrix (ECM) remodeling, fostering a permissive milieu for tumor cell proliferation and dissemination, while concurrently modulating key signaling cascades implicated in oncogenic processes (Ping et al., 2021).

The clinical significance of CAFs is underscored by their association with adverse prognostic outcomes across diverse cancer types, signifying their potential as prognostic biomarkers (Min et al., 2021; Mak et al., 2022; Xu et al., 2023). Their intricate interplay with cancer cells and stromal constituents not only influences tumor behavior but also dictates therapeutic responses, often facilitating the emergence of chemoresistance. A comprehensive understanding of the reciprocal crosstalk between CAFs and tumor cells has thus prompted endeavors to devise targeted therapeutic interventions aimed at disrupting tumor-stroma interactions, with CAFs emerging as promising targets for circumventing therapeutic resistance and augmenting treatment efficacy (Zhang et al., 2023).

Nevertheless, the heterogeneity inherent to CAF populations poses a formidable challenge in delineating their precise functional roles and devising tailored therapeutic strategies (Zhang et al., 2023). Recent advancements in single-cell transcriptomic profiling, notably through technologies such as single-cell RNA sequencing (scRNA-seq) and spatial transcriptomics, have unveiled a spectrum of CAF subpopulations characterized by distinct gene expression profiles, functional attributes, and spatial distribution within the TME (Zhang et al., 2020; Kieffer et al., 2020; Wang et al., 2021; Hu et al., 2022; Luo et al., 2022; Liu et al., 2023; Cords et al., 2023). These discrete CAF subsets exhibit nuanced phenotypic characteristics and exert context-dependent effects on tumor progression, necessitating a nuanced approach towards therapeutic targeting.

In this review, we systematically interrogate the landscape of CAF heterogeneity by leveraging scRNA-seq data encompassing diverse malignancies. In total, we reviewed and systematized data from 36 scRNA-seq studies, encompassing 17 types of human malignant tumors (Elyada et al., 2019; Zhang et al., 2020; Chen et al., 2020; Dominguez et al., 2020; Kieffer et al., 2020; Lin et al., 2020; Wang et al., 2021; Zhang et al., 2021; Che et al., 2021; Chen et al., 2021; Galbo et al., 2021; Hornburg et al., 2021; Olbrecht et al., 2021; Pu et al., 2021; Li et al., 2022; Bischoff et al., 2022; Li et al., 2022; Chen et al., 2022; Li et al., 2022; Deng et al., 2022; Giguelay et al., 2022; Hu et al., 2022; Loret et al., 2022; Luo et al., 2022; Obradovic et al., 2022; Yang et al., 2022; Zhao et al., 2022; Liu et al., 2023; Qin et al., 2023; Liu et al., 2023; Zhang et al., 2023; Cords et al., 2023; Ma et al., 2023; Pan et al., 2023; Wang et al., 2023; Werba et al., 2023). As a result, we identified and characterized three primary CAFs populations: myofibroblast-like CAFs, inflammatory CAFs, and antigen-presenting CAFs. We delineated their unique gene signatures, which allows to distinguish each CAF population from other CAFs and TME cells. Notably, our findings elucidate previously overlooked genes implicated in CAF function, thereby enriching our understanding of CAF biology. Additionally, we discussed the presence of rare CAF subtypes characterized in only a few studies.

Furthermore, we scrutinize the role of distinct CAF subpopulations in mediating tumor resistance to conventional and targeted therapeutic modalities. By elucidating the differential impact of CAF heterogeneity on treatment responses, we emphasize the need to integrate CAF profiling into therapeutic decision-making processes. Our synthesis not only underscores the complexity of the TME but also informs the rational design of anti-CAF therapies tailored to target specific tumor-promoting CAF populations, thereby heralding a paradigm shift towards precision medicine in cancer therapeutics.

## Heterogeneity of CAFs

Recent studies have unveiled the heterogeneous nature of CAFs, shedding light on the existence of diverse cellular populations within this entity (Sugimoto et al., 2006; Öhlund et al., 2017). This diversity for the first time was noted by the identification of two distinct subpopulations of CAFs within breast and pancreatic carcinomas, distinguished by the expression patterns of the S100A4 gene (Sugimoto et al., 2006). Further investigations, both *in vitro* and *in vivo*, have underscored the complexity of CAFs, revealing spatially separated, phenotype reversible, and mutually exclusive subtypes within co-cultures of murine pancreatic stellate cells, the main source of CAFs within pancreatic cancer, and pancreatic ductal adenocarcinoma organoids (Öhlund et al., 2017).

However, elucidating the specific subtypes of CAFs in tumor tissues, alongside their unique transcriptional characteristics, remained challenging until the emergence of scRNA-seq technologies. Despite limitations inherent to scRNA-seq, such as its reduced sensitivity to transcripts with low expression levels, this approach offers unparalleled advantages in dissecting the heterogeneity of tissue cell populations (Zhang et al., 2021).

Unlike traditional methods, scRNA-seq analysis eliminates the necessity to pre-isolate fibroblast populations using specific protein markers. Instead, it enables a comprehensive analysis of all cellular constituents within tissues. Additionally, it overcomes obstacles associated with cell culturing, which has been shown to potentially alter CAF phenotype over prolonged periods (Fuyuhiro et al., 2011; Wang et al., 2015; Sahai et al., 2020; Ping et al., 2021). For example, gastric CAFs had increased expression of standard CAF marker *ACTA2* compared to normal fibroblasts at the third passage, but by the eighth passage, they did not demonstrate significant difference in the expression of this gene (Fuyuhiro et al., 2011). Thus, scRNA-seq emerges as a practical alternative for exploring CAF heterogeneity.

**FIGURE 1:**
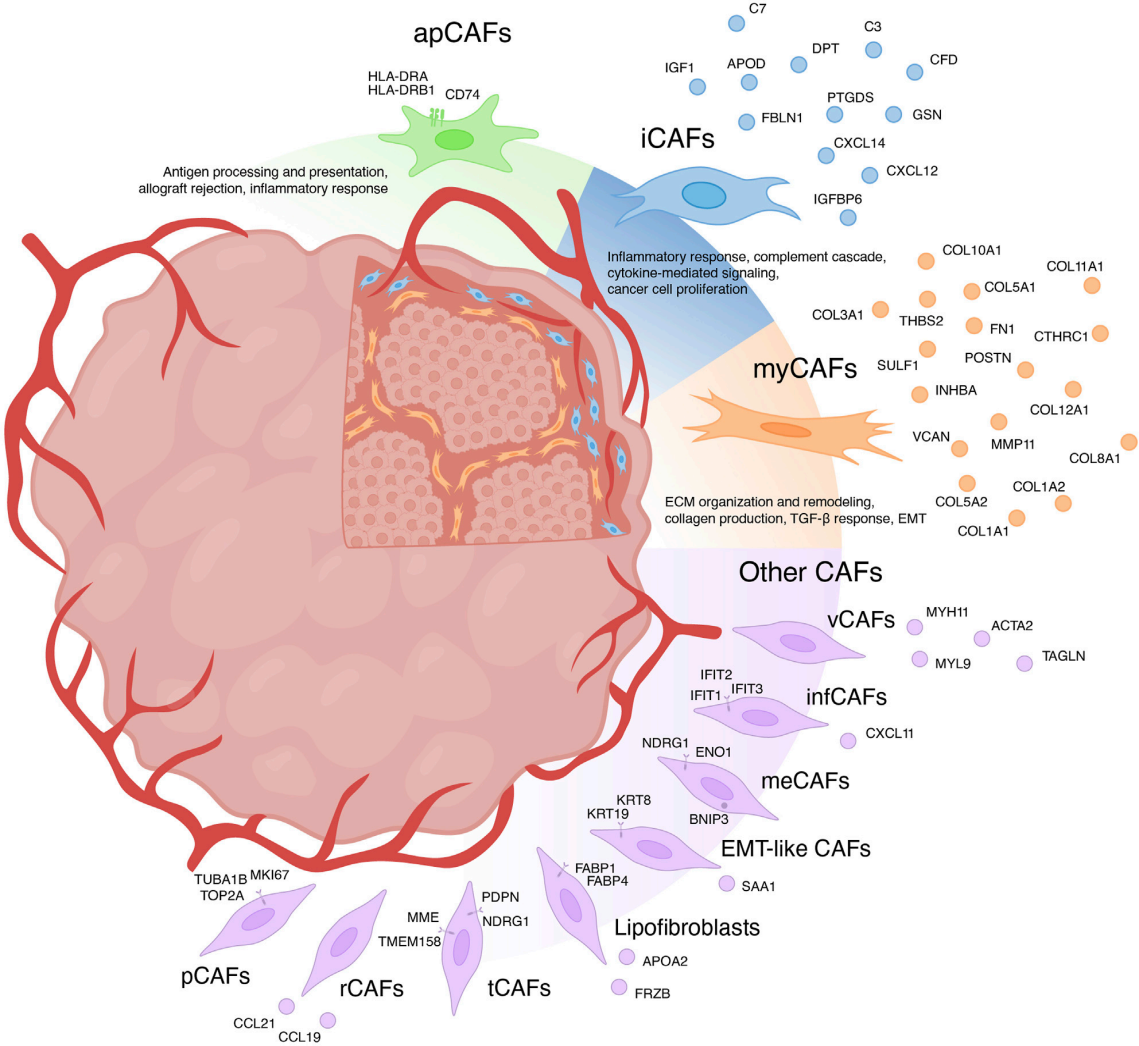
Heterogeneity of cancer-associated fibroblasts (CAFs) and their markers. myCAFs, myofibroblasts-like CAFs; iCAFs, inflammatory CAFs; apCAFs, antigen-presenting CAFs; pCAFs, proliferative CAFs; ifnCAFs, interferon-response CAFs; meCAFs, metabolic CAFs; EMT-like CAFs, epithelial to mesenchymal transition-like CAFs; tCAFs, tumor-like CAFs; rCAFs, reticular-like CAFs; vCAFs, vascular CAFs.

For a long time, CAFs were believed to consist only of two distinct populations: myofibroblasts-like CAFs (myCAFs) and inflammatory CAFs (iCAFs) (Sugimoto et al., 2006; Öhlund et al., 2017). With the advent of scRNA-seq, the existence of multiple phenotypically and functionally distinct CAF populations has become apparent (Figure 1) (Galbo et al., 2021; Cords et al., 2023; Ma et al., 2023). Currently, there is growing evidence supporting the existence of another CAF population in each tumor type—antigen-presenting CAFs (apCAFs) (Figure 1) (Zhang et al., 2020; Kieffer et al., 2020; Wang et al., 2021; Hu et al., 2022; Luo et al., 2022; Liu et al., 2023; Cords et al., 2023). Moreover, scRNA-seq studies have identified other rarely occurring CAF populations in the microenvironment of different tumor types (Figure 1) (Zhang et al., 2020; Galbo et al., 2021; Li et al., 2022; Cords et al., 2023; Ma et al., 2023). The characteristics of each CAF subpopulation will be further discussed in detail.

## Myofibroblasts-like subtype of cancer-associated fibroblasts—myCAFs

### Gene signature of myCAF

Initially, myCAFs were discerned from pancreatic ductal adenocarcinoma, delineated as contractile matrix-producing fibroblasts (FAP+ αSMAhigh CAFs), showcasing heightened expression of *ACTA2* and TGFβ response genes including *CTGF* and *COL1A1* (Öhlund et al., 2017). The advent of scRNA-seq has uncovered a similar fibroblast phenotype across diverse solid human tumors. However, transcriptional profiles attributed to myCAFs exhibit variations across studies. Broadly, myCAFs characterization via scRNA-seq can be grouped into two categories.

In the first category, myCAFs are distinguished as a distinct cell population typified by elevated expression of the blood vessel wall gene *RGS5*, contractility genes (*MYH11*, *ACTA2*, *TAGLN*, *MYL9*), and other pericyte markers (*NDUFA4L2*, *ADIRF*) (Elyada et al., 2019; Chen et al., 2020; Galbo et al., 2021; Pu et al., 2021; Li t al., 2022; Chen et al., 2022; Hu et al., 2022; Luo et al., 2022; Obradovic et al., 2022; Yang et al., 2022; Zhao et al., 2022; Liu et al., 2023; Qin et al., 2023). Functionally, these cells are implicated in processes such as angiogenesis, smooth muscle contraction, and vascular wound healing. Apart from being referred to as myCAFs, they are also labeled as contractile CAFs (Che et al., 2021; Giguelay et al., 2022), stellate-like CAFs (Wang et al., 2021), MCAM+ CAFs (Wang et al., 2023), RGS5+ CAFs (Olbrecht et al., 2021; Zhang et al., 2023) and MYH11+ CAFs (Olbrecht et al., 2021). However, evidence suggests that a similar transcriptional profile is characteristic of pericytes and other mural cells (van Splunder et al., 2024). Consequently, several scRNA-seq investigations propose categorizing this population as pericytes rather than fibroblasts (Lin et al., 2020; Zhang et al., 2021; Bischoff et al., 2022; Li et al., 2022; Cords et al., 2023; Werba et al., 2023). Furthermore, scRNA-seq analysis of normal muscular organs discerned a 90-gene signature capable of distinguishing fibroblasts from mural cells (Muhl et al., 2020). Consistent with this, heightened expression of genes including *MYL9*, *MYH11*, *MCAM*, *TAGLN*, and *ACTA2* suggests affiliation with mural cells. This raises the question: does a distinct population of RGS5 + MYH11+ CAFs truly exist or they should be considered as pericytes or other mural cells? Recent studies suggest that the analysis of scRNA-seq data allows to separate these two similar types of cells within tumors: pericytes and vascular CAFs (vCAFs), likely originating from vascular cells (Cords et al., 2023). Notably, vCAFs, akin to pericytes, exhibit elevated expression of angiogenesis-related genes (*MYH11*, *ACTA2*, *MCAM*, and *ADIRF*) (Cords et al., 2023). However, *RGS*5 gene expression is much lower in vCAFs compared to pericytes. Similar to pericytes, vCAFs localize around endothelial cells within vascular structures. Nevertheless, accurate classification of all RGS5 + MYH11+ cell populations is imperative for further exploration of vascular tumor cells and CAFs.

The second category of myCAFs, also termed as such in studies, is typified by expression of collagen-encoding genes (*COL1A1*, *COL10A1*, *COL11A1*, *CTHRC1*, etc.), matrix metalloproteinases (*MMP11*), non-collagenous ECM genes (*POSTN*, *THBS2*, and *VCAN*, *FN1*), and signal transduction genes (*SULF1*, *INHBA*) (Kieffer et al., 2020; Lin et al., 2020; Wang et al., 2021; Bischoff et al., 2022; Loret et al., 2022; Liu et al., 2023; Werba et al., 2023). These fibroblasts, also referred to as matrix CAFs (mCAFs) (Zhang et al., 2020; Cords et al., 2023; Ma et al., 2023), ECM-remodeling CAFs (Che et al., 2021; Giguelay et al., 2022), desmoplastic CAFs (dCAFs) (Galbo et al., 2021), TGF-β-activated CAFs (TGFB CAFs) (Hornburg et al., 2021), classical CAFs (cCAFs) (Chen et al., 2021), and others (Olbrecht et al., 2021; Deng et al., 2022; Pan et al., 2023), are intricately associated with ECM organization and remodeling, collagen production, TGF-β response and pathways governing the epithelial to mesenchymal transition (EMT). Studies suggest that these fibroblasts arise from resident fibroblasts and are localized within collagen-rich regions of the tumor (Bartoschek et al., 2018; Zhang et al., 2020; Cords et al., 2023). The upregulation of genes linked to TGF-β-induced reactive stroma implies a pivotal role for TGF-β in activating resident fibroblasts and shaping the myCAF phenotype (Bates et al., 2015; Biffi et al., 2019; Kieffer et al., 2020; Hornburg et al., 2021; Giguelay et al., 2022). Importantly, TGF-β1-activated fibroblasts accelerate cancer cell motility and invasion (Yeung et al., 2013; Huang et al., 2021; Yoon et al., 2021). The localization of matrix-associated myCAFs at the invasive tumor front further underscores their role in tumor invasion (Zhang et al., 2020).

The variability in characteristics among myCAFs contributes to ambiguity when comparing results across different studies. Hence, caution is warranted in analyzing the RGS5+MYH11+ cell population within tumors, emphasizing the necessity to confirm whether this population represents pericytes. Given that the term myCAFs was initially coined for fibroblasts capable of producing a collagen matrix, we propose categorizing the second group of cells as myCAFs.

### Prognostic potential of myCAFs markers

The expression of markers characteristic of myCAFs serves as a distinguishing feature among various CAF populations. However, the specificity of this gene signature to myCAFs amidst all tumor cell populations remains a subject of inquiry. scRNA-seq studies indicate that the expression of most myCAFs marker genes is primarily confined to fibroblasts within the tumor milieu (Lambrechts et al., 2018; Elyada et al., 2019; Chen et al., 2020; Wang et al., 2021; Zhang et al., 2021; Che et al., 2021; Olbrecht et al., 2021; Pu et al., 2021; Luo et al., 2022; Zhao et al., 2022; Qin et al., 2023; Zhang et al., 2023; Ma et al., 2023; Wang et al., 2023; Werba et al., 2023). Furthermore, analysis of immunohistochemistry staining (IHC) images from the Human Protein Atlas database (Uhlen et al., 2017) reveals predominant localization of proteins such as COL3A1, POSTN, VCAN, FN1, COL12A1 in the stroma of solid tumor types (Supplementary Figure S1). These findings suggest that the myCAF gene signature is not characteristic of any other cell population within the tumor and its microenvironment. Consequently, alterations in the expression of this gene signature in tumor tissues likely reflect changes in the abundance of the myCAF population.

**FIGURE 2:**
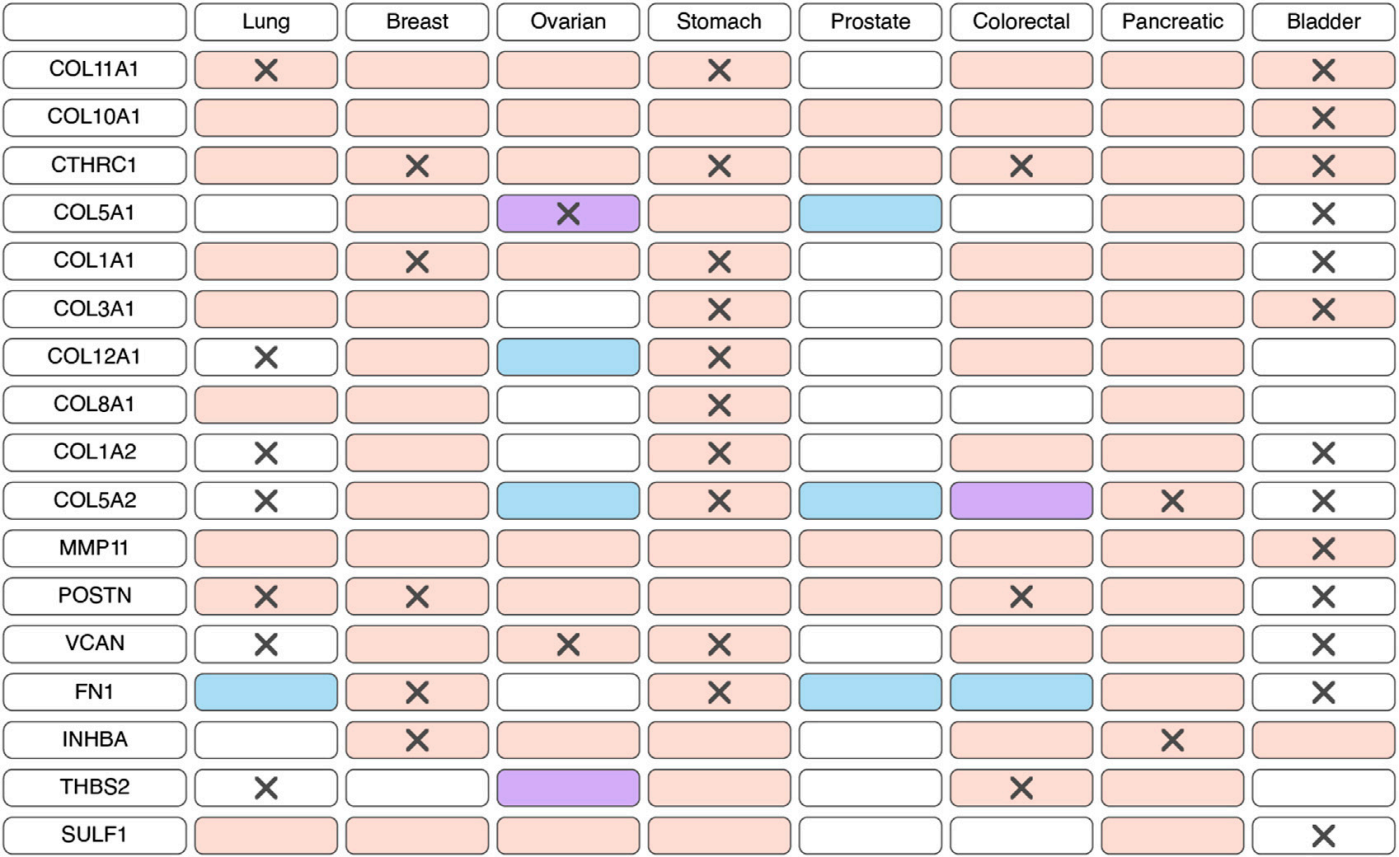
Changes in the expression and prognostic significance of myofibroblast-like cancer-associated fibroblasts (myCAFs) markers across different types of tumors. Red indicates increased expression of the gene/protein in the tumor compared to normal tissue, blue indicates decreased expression, and purple indicates both increased and decreased expression observed in different studies. A cross symbol indicates an association of increased gene expression with poor prognosis, while a checkmark indicates an association with good prognosis.

According to bulk RNAseq analyses, myCAF markers are predominantly upregulated at transcriptional level in tumor tissues compared to normal tissues across various cancer types (Figure 2; Supplementary Table S1). These observations are further substantiated at the protein level through comparisons of IHC staining patterns in tumor and normal tissues (Figure 2; Supplementary Table S1). Notably, the myCAF gene signature exhibits a significant correlation with poor survival outcomes among patients diagnosed with stomach adenocarcinoma, kidney carcinoma, mesothelioma, low-grade glioma, cervical squamous cell carcinoma, bladder urothelial carcinoma, skin cutaneous melanoma, lung carcinoid tumors and prostate cancer (Galbo et al., 2021; Bischoff et al., 2022; Liu et al., 2023; Pan et al., 2023). Furthermore, elevated expression of most myCAFs marker genes correlates with poor overall survival across various cancer types (Figure 2; Supplementary Table S2). Thus, the heightened presence of the myCAF population emerges as a characteristic feature of tumor tissues and is associated with an unfavorable prognosis.

### Contribution of myCAFs to the development of chemoresistance

**FIGURE 3:**
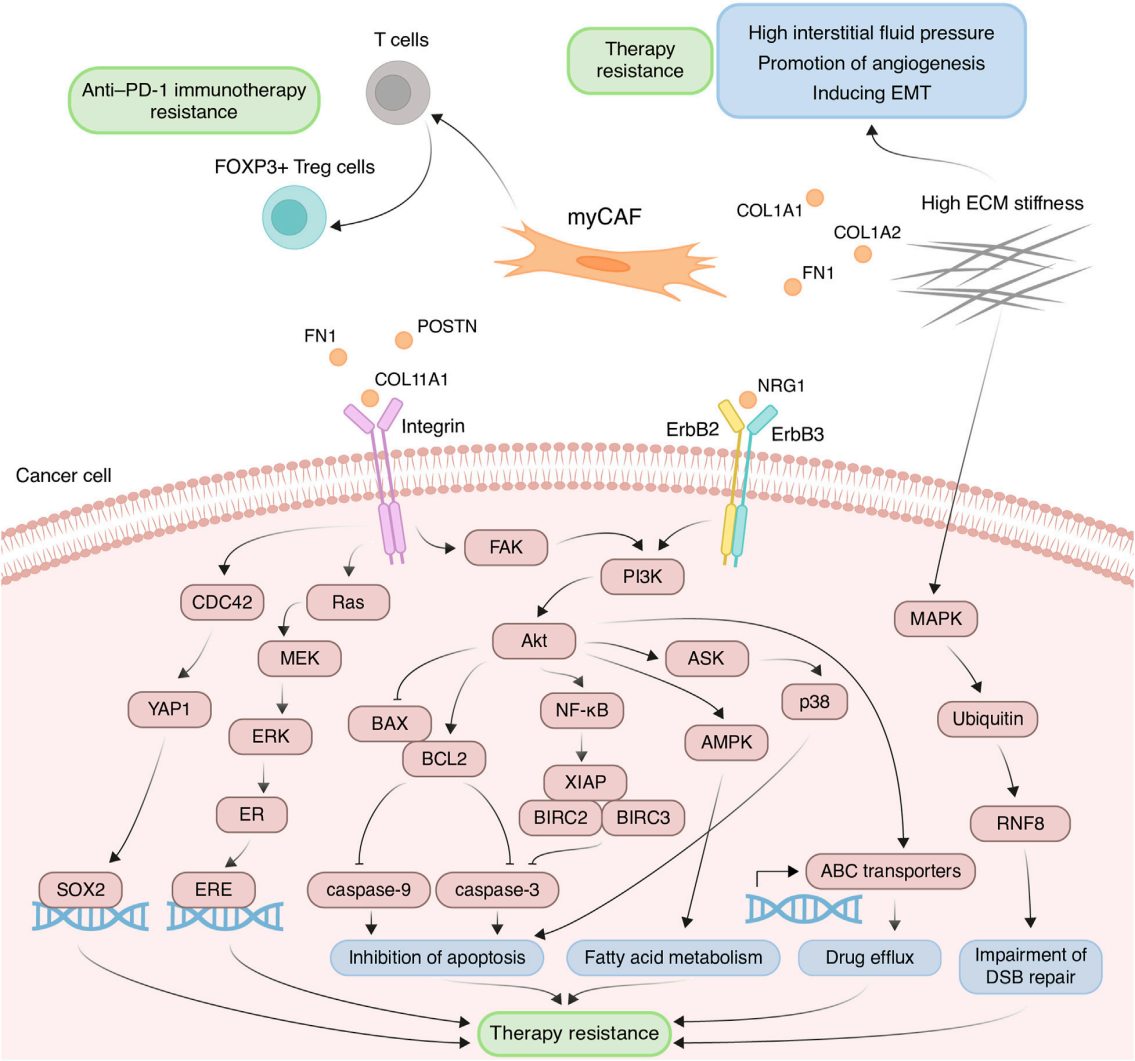
Principal roles and related mechanisms of myofibroblasts-like cancer-associated fibroblasts (myCAFs) in the development of therapeutic resistance of tumors. EMT, epithelial to mesenchymal transition; ECM, extracellular matrix; DSB, double-strand break.

The myCAF population plays a pivotal role within the tumor stromal microenvironment, actively participating in the remodeling of the ECM, a process intricately linked to the development of chemoresistance. One such modification characteristic of tumor tissues is ECM stiffening, primarily caused by the increased deposition of type I collagen and fibronectin (Cox and Erler, 2011). These proteins are coded by genes *COL1A1*, *COL1A2*, and *FN1*, primarily expressed in myCAFs. This stiffening of the ECM poses significant challenges to effective capillary transport of therapeutic agents into tumor tissues, owing to the resultant elevation in interstitial fluid pressure (Figure 3) (Stylianopoulos et al., 2018). Furthermore, myCAF-mediated ECM stiffness serves as a driver of angiogenesis, further fueling tumor progression (Figure 3). Inhibition of fibroblast activity using renin-angiotensin system inhibitors results in decreased ECM stiffness and enhances the anti-angiogenic effects of bevacizumab in patients with metastatic colorectal cancer (Shen et al., 2020). Notably, in various cancers such as pancreatic and breast cancer, the heightened ECM stiffness contributes to resistance against chemotherapeutic agents like paclitaxel and doxorubicin, respectively, through the induction of EMT processes (Figure 3) (Rice et al., 2017; Joyce et al., 2018). Moreover, the rigidity of the ECM may be linked to the regulation of DNA double-strand break repair processes, thereby influencing cellular sensitivity to genotoxic agents, including etoposide and cisplatin (Deng et al., 2020). In breast cancer cells growing on low stiffness ECM, the activation of MAP4K4/6/7 kinases and subsequent ubiquitin phosphorylation occurs. This leads to the impairment of RNF8-mediated ubiquitin signaling and double-stranded DNA repair deficiency (Figure 3) (Deng et al., 2020).

Another critical mechanism underlying drug resistance, facilitated by myCAFs, involves the integrin-mediated adhesion of tumor cells to ECM components, reinforcing linkages between integrins and the cytoskeleton, consequently leading to elevated formation of focal adhesions (Valdembri and Serini, 2021). The serine/threonine kinase Akt signaling pathway in cancer cells emerges as a key player in this integrin-mediated resistance mechanism. ECM proteins produced by myCAFs, such as collagens, POSTN and FN1, interact with integrins on the surface of tumor cells, thereby activating Akt signaling (Figure 3) (Elango et al., 2022; Wang et al., 2022; Sun et al., 2023). For instance, co-cultivation of ovarian cancer cells with CAFs or on the plates pre-coated with COL11A1 attenuates cisplatin-induced apoptosis (Rada et al., 2018; Nallanthighal et al., 2020). This effect is achieved by activating the Akt signaling pathway through the interaction of COL11A1 with DDR2 receptor tyrosine kinase and α1β1 integrin on the cell surface of ovarian cancer cells. In turn, Akt activates downstream NF-kB signaling, leading to the induction of the expression of three antiapoptotic proteins, including XIAP, BIRC2, and BIRC3, and decreasing levels of cleaved caspase-3 (Figure 3) (Rada et al., 2018). Moreover, Akt signaling leads to the phosphorylation of 5′ AMP-activated protein kinase and upregulates mitochondrial fatty acid oxidation, which is necessary for maintaining ovarian cancer cell survival (Figure 3) (Nallanthighal et al., 2020).

Another downstream mechanism activated by Akt leads to the upregulation of the antiapoptotic protein BCL-2 and the downregulation of proapoptotic protein BAX. Altered balance between BCL-2 and BAX suppressed the apoptotic program by reduction in cleaved caspase-3/9 expression (Figure 3). This mechanism mediates the resistance of pancreatic cancer cells cultivated on COL11A1-coated plates to gemcitabine (Wang et al., 2021) and the resistance of ovarian cancer cells exposed to recombinant POSTN or co-cultured with POSTN-overexpressing fibroblasts to cisplatin (Chu et al., 2020). Furthermore, FN1/PI3K/Akt signaling contributes to breast cancer cell resistance against docetaxel by inhibiting the ASK1/p38 apoptotic pathway (Figure 3) (Xing et al., 2008). Activation of PI3K/Akt/αvβ5 signaling axis by unfolded type III domain of FN1 protects non-small cell lung cancer cells from apoptosis induced by TNF-related apoptosis inducing ligand (Cho et al., 2016).

ECM protein-integrin binding can activate other signaling cascades besides Akt in tumor cells. For instance, FN1 binding to β1 integrin on colorectal cancer cells activates FAK/CDC42/YAP signaling, leading to upregulation of the transcription factor SOX2 and consequent resistance to 5-fluorouracil and cisplatin (Figure 3) (Ye et al., 2020). Additionally, stromal FN1 promotes both FAK/RAS/MEK/ERK and FAK/PI3K/Akt pathways in breast cancer cells, leading to estrogen receptor-α phosphorylation and subsequent tamoxifen sensitivity (Figure 3) (Pontiggia et al., 2012). Furthermore, the interaction of ECM proteins with integrins significantly impacts the activity of ATP binding cassette (ABC) transporters, which recognize cytostatic drugs and export them from the cytosol. Cultivating breast cancer cells on the microplates coated with collagen I and fibronectin leads to enhanced resistance to doxorubicin, cisplatin, and mitoxantrone, due to increased activity of ABC efflux transporters (Figure 3) (Baltes et al., 2020).

myCAFs also contribute to anti-cancer therapy resistance through mechanisms beyond ECM remodeling. For instance, myCAFs enhance proliferation, migration, and resistance to carboplatin and cisplatin in cervical squamous cell carcinoma via the NRG1/ERBB3 pathway activation (Figure 3) (Liu et al., 2023). Furthermore, the expression of the myCAF-specific gene signature correlates with poor response to anti-PD-1 immunotherapy in patients with urothelial carcinoma, melanoma, and non-small cell lung cancer, potentially mediated by myCAF-induced modulation of the immune microenvironment (Kieffer et al., 2020; Pan et al., 2023). The impact on the effectiveness of anti–PD-1 immunotherapy may be explained by the ability of myCAFs *in vitro* to increase the proportion of CD4^+^ CD25^+^ FOXP3^+^ Tregs among CD4^+^CD25^+^ T lymphocytes and elevate the levels of PD-1 and CTLA4 proteins on the surface of CD4^+^ CD25^+^ FOXP3^+^ Tregs (Figure 3) (Kieffer et al., 2020).

In summary, the myCAF population serves not only as a prognostic indicator but also as a promising therapeutic target. Other myCAF markers, including INHBA, SULF1, CTHRC1, VCAN, and COL5A1, have also been implicated in influencing resistance to anticancer drugs (Table 1). A significant limitation of these studies is the use of tumor cells for investigation of myCAFs markers in the development of resistance to various anti-tumor medications. Given that this gene signature is characteristic of CAFs, not tumor cells, conducting similar studies using CAFs could provide greater clarity on acquired chemoresistance in tumors.

**TABLE 1:** The role of myofibroblasts-like cancer-associated fibroblast markers in the development of therapeutic resistance of tumors.

Gene	Tumor type	Therapy	Effect/Mechanism	References
VCAN	ovarian cancer (patients tumor tissues)	platinum-based adjuvant chemotherapy	High-level of VCAN expression is associated with a worse therapy response	Ghosh et al. (2010)
SULF1	ovarian cancer (*in vitro*, A2780 and SKOV3)	cisplatin	Knockdown of SULF1 in cancer cells leads to decreased resistance	Ouyang et al. (2019)
SULF1	ovarian cancer (*in vitro*, OV167 and OV202)	cisplatin	Knockdown of SULF1 in cancer cells leads to increased resistance	Staub et al. (2007)
COL11A1	ovarian cancer (*in vitro*, ES2)	cisplatin	Cultivation cancer cells on COL11A1-coated plates leads to increased resistance/Activation of XIAP, BIRC2, BIRC3 expression leads to a subsequent inhibition of downstream caspase 3	Rada et al. (2018)
COL11A1	ovarian cancer (*in vitro*, ES2)	cisplatin	Cultivation cancer cells on COL11A1-coated plates leads to increased resistance/Activation of the Src/Akt/AMPK pathways	Nallanthighal et al. (2020)
COL11A1	ovarian cancer (*in vitro*, A2780 and *in vivo*, mice xenografts)	cisplatin and paclitaxel	Overexpression of COL11A1 in tumor cells leads to increased resistance/Activation of IKKβ/NF-κB/TWIST1,Mcl-1,GAS6	Wu et al. (2017), Wu et al. (2019)
COL5A1	ovarian cancer (*in vitro*, OVCAR3)	paclitaxel	Knockdown of COL5A1 in cancer cells leads to decreased resistance	Zhang et al. (2021d)
COL11A1	ovarian cancer (*in vitro*, SKOV3)	radiotherapy	Knockdown of COL11A1 in cancer cells leads to decreased resistance/Deactivation of the Akt pathway	Zhu et al. (2021)
FN1	breast cancer (*in vitro*, MDA-MB-231)	docetaxel	Cultivation of cancer cells on FN1-coated plates leads to increased resistance/Activation of the Akt2/ASK1/p38 pathway	Xing et al. (2008)
FN1	breast cancer (*in vitro*, LM05-E and MCF-7)	tamoxifen	Cultivation of cancer cells on FN1-coated plates leads to increased resistance/Activation of the PI3K/AKT and MAPK/ERK1/2 pathways	Pontiggia et al. (2012)
MMP11	breast cancer (MDA-MB-231 and MCF-7)	lapatinib	Knockdown of Circ-MMP11 in cancer cells leads to decreased resistance/Increase in ANLN expression through prevention of miR-153-3p sponging	Wu et al. (2021)
INHBA	breast cancer (*in vitro*, 21-MT1)	lapatinib	Knockdown of INHBA in cancer cells leads to decreased resistance	Liu et al. (2022)
FN1	breast cancer (*in vitro*, MDA-MB-231)	doxorubicin	Cultivation of cancer cells on FN1-coated plates leads to increased resistance/Activation of the FAK/Src pathway	Assidicky et al. (2022)
COL1	breast cancer (*in vitro*, MDA-MB-231)	doxorubicin, mitoxantrone	Cultivation cancer cells on COL1-coated plates leads to increased resistance/Increase in ABC transporter activity and expression	Baltes et al. (2020)
COL1A2	gastric cancer (*in vitro*, AGS and MKN-45)	apatinib	Knockdown of COL1A2 in cancer cells leads to decreased resistance Overexpression of COL1A2 in cancer cells leads to increased resistance	Yu et al. (2022)
POSTN	gastric cancer (patients tumor tissues)	immune checkpoint blockade therapy	High-level of POSTN expression is associated with a worse therapy response	You et al. (2023)
VCAN	gastric cancer (patients tumor tissues)	adjuvant chemotherapy, adjuvant chemoradiotherapy and immune checkpoint blockade therapy	High-level of VCAN expression is associated with a worse therapy response	Song et al. (2022)
POSTN	gastric cancer (*in vitro*, SW1990 and Panc-1; mice tumor xenografts)	gemcitabine	Cultivation of cancer cells with recombinant periostin leads to increased resistance/Activation of the Akt and Erk pathways	Liu et al. (2016)
POSTN	non-small cell lung cancer (*in vivo*, mice xenografts of HCC4006 and CAFs)	Osimertinib	Knockdown of POSTN in CAFs leads to increased resistance	Takatsu et al. (2023)
FN1	non-small cell lung cancer (*in vitro*, A549 and H1299)	cetuximab, irradiation	FN1 knockdown leads to decreased resistance	Eke et al. (2013)
COL11A1	non-small cell lung carcinoma (*in vitro*, H520 and H23)	cisplatin	Overexpression of COL11A1 in tumor cells leads to increased resistance/Activation of the Smad pathway	Shen et al. (2016)
POSTN	lung cancer (*in vitro*, A549)	cisplatin	Overexpression of POSTN in cancer cells leads to increased resistance/Activation of the Stat3/survivin and Akt/survivin pathways	Hu et al. (2016)
FN1	non-small cell lung cancer (*in vitro*, A549 both sensitive and cisplatin-resistant)	cisplatin	FN1 knockdown leads to decreased resistance/Suppression of the Wnt/β-catenin signaling pathway	Gao et al. (2016)
FN1	lung cancer (*in vitro*, NCI-H460)	TNF-related apoptosis inducing ligand	Обработка cancer cells with FnIII-1c (FN1 fragment) leads to increased resistance/Activation of the PI3K/Akt/αvβ5 pathway	Cho et al. (2016)
POSTN	colon cancer (*in vitro*, HCT116 and LoVo)	5-fluorouracil	Knockdown of POSTN in cancer cells leads to decreased resistance	Liu et al. (2021)
POSTN	colon cancer (*in vitro*, SW480 and HT-29)	oxaliplatin, 5-fluorouracil	Overexpression of POSTN in cancer cells leads to increased resistance/Activation of the PI3K/Akt/survivin pathway	Xiao et al. (2015)
FN1	colorectal cancer (*in vitro*, HCT116 and SW480, mice tumor xenografts)	5-fluorouracil, cisplatin	Cultivation of cancer cells with FN1 leads to increased resistance/Activation of the CDC42/YAP-1/SOX2 pathway	Ye et al. (2020)
INHBA	pancreatic cancer (data from NCI-60 database)	Zoledronate and Dasatinib	High-level of INHBA expression is associated with a worse therapy response	Li et al. (2023)
COL11A1	pancreatic cancer (*in vitro*, BxPC-3)	gemcitabine	Cultivation cancer cells on COL11A1-coated plates leads to increased resistance/Activation of the Akt/CREB pathway leads to a subsequent decrease in the BAX/BCL-2 ratio and the inhibition of downstream caspase 3 and caspase 9	Wang et al. (2021b)
COL1	pancreatic cancer (*in vitro*, Panc1 and CD18/HPAF-II)	gemcitabine	Cultivation of cancer cells in 3D collagen gels leads to increased resistance/H3K9 and H3K27 acetylation and activation of p300, PCAF, GCN5 HAT expression	Dangi-Garimella et al. (2013)
FN1	pancreatic cancer (*in vitro*, AsPC-1, BxPC-3, Capan-2, HPAF-II, MIA PaCa-2, PANC-1 и SW-1990)	gemcitabine	Cultivation of cancer cells with pancreatic stellate cells-derived FN1 leads to increased resistance	Amrutkar et al. (2019)
INHBA	Head and neck squamous cell carcinoma (*in vitro*, SVpgC2a, SVFN8 и CaLH2)	cisplatin, 5-fluorouracil, paclitaxel, docetaxel	Knockdown of INHBA in cancer cells leads to decreased resistance	Khera et al. (2023)
FN1	bladder cancer (*in vitro*, T24)	mitomycin C	Cultivation of cancer cells on FN1-coated plates leads to increased resistance/Activation of the PI3-K/Akt pathway	Pan et al. (2009)
FN1	osteosarcoma (*in vitro*, MG63, KH-OS)	doxorubicin	Knockdown of FN1 in cancer cells leads to decreased resistance Overexpression of FN1 in cancer cells leads to leads to increased resistance	Kun-Peng et al. (2019)
COL1A1	hepatocellular carcinoma (*in vitro*, Huh7 spheroids)	sorafenib, cisplatin	Knockdown of COL1A2 in cancer cells leads to decreased resistance	Song et al. (2017)
COL1A1	cervical cancer (*in vitro*, HeLa and CaSki)	radiotherapy	Overexpression of COL11A1 in cancer cells leads to increased resistance/Activation of the PI3K/AKT pathway leads to a subsequent decrease in the BAX/BCL-2 ratio and the inhibition of downstream caspase 3	Liu et al. (2017b)
COL1A1	Nasopharyngeal carcinoma (*in vitro*, CNE-2R, and patients data)	radiotherapy	Knockdown of COL1A1 leads to decreased resistance The expression level of COL1A1 is elevated in radioresistant tumors compared to radiosensitive tumors	Guo et al. (2019)

## Inflammatory subtype of cancer-associated fibroblasts—iCAFs

### Gene signature of iCAF

It is widely recognized that CAFs play a crucial role in shaping the unique tumor immune microenvironment, facilitating tumor immune evasion (Mhaidly and Mechta-Grigoriou, 2021). Among the diverse CAF subtypes, iCAFs stand out for their distinct immunomodulatory secretory profile (Figure 1). iCAFs are characterized by their expression of various genes, including members of the chemokine ligand family (*CXCL12* and *CXCL14*), the insulin-like growth factor family (*IGF1* and *IGFBP6*), complement-regulatory genes (*C3*, *C7,* and *CFD),* and some other genes (*APOD*, *FBLN1*, *PTGDS*, *DPT,* and *GSN*) (Elyada et al., 2019; Zhang et al., 2020; Chen et al., 2020; Wang et al., 2021; Galbo et al., 2021; Pu et al., 2021; Li et al., 2022; Chen et al., 2022; Li et al., 2022; Hu et al., 2022; Loret et al., 2022; Obradovic et al., 2022; Yang et al., 2022; Liu et al., 2023; Cords et al., 2023; Ma et al., 2023; Pan et al., 2023; Werba et al., 2023).

The functions attributed to iCAFs encompass a broad spectrum, ranging from involvement in inflammatory responses and complement cascades to mediating cytokine-driven signaling and fostering cancer cell proliferation. Unlike myCAFs, iCAFs are located more distant from cancer cells (Öhlund et al., 2017; Wu et al., 2020). It has also been shown that iCAFs encircle vCAFs and the endothelial cells lining the tumor blood vessels (Cords et al., 2023). Notably, the induction of the iCAF phenotype predominantly occurs through the IL1-IL1R1 axis and can occur independently of direct contact with cancer cells (Biffi et al., 2019), leading researchers to frequently refer to them as IL1+ CAFs in scientific investigations (Dominguez et al., 2020; Hornburg et al., 2021).

### Prognostic potential of iCAFs markers

**FIGURE 4:**
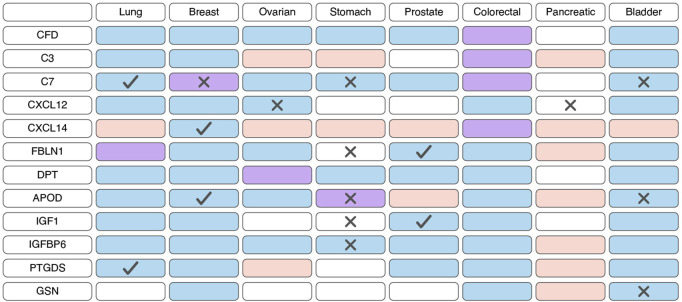
Changes in the expression and prognostic significance of inflammatory cancer-associated fibroblasts (iCAFs) markers across different types of tumors. Red indicates increased expression of the gene/protein in the tumor compared to normal tissue, blue indicates decreased expression, and purple indicates both increased and decreased expression observed in different studies. A cross symbol indicates an association of increased gene expression with poor prognosis, while a checkmark indicates an association with good prognosis.

Based on scRNA-seq data, iCAFs, as well as myCAFs, exhibit a distinct gene signature, enabling their differentiation from other tumor cell populations (Lambrechts et al., 2018; Chen et al., 2020; Wang et al., 2021; Luo et al., 2022; Liu et al., 2023; Qin et al., 2023; Wang et al., 2023). Consequently, alterations in the expression of this gene signature within tumor tissues are indicative of shifts in the abundance of iCAFs. Unlike myCAFs markers, markers associated with iCAFs tend to exhibit significant downregulation at the transcriptional level in tumor tissue compared to normal tissue across various cancer types (Figure 4; Supplementary Table S3). However, an exception to this trend is observed in pancreatic cancer, where the expression of most iCAF markers notably increases in tumor tissue (Figure 4; Supplementary Table S3). Additionally, the expression of certain genes correlates with overall survival across different tumor types (Figure 4; Supplementary Table S4).

Similar to myCAFs, iCAFs are also mentioned in the context of disease prognosis; however, these findings are contradictory. Higher iCAF abundance has been associated with poor clinical outcomes and diminished response to immunotherapy in patients with bladder cancer, low-grade glioma, and head and neck squamous cell carcinoma (Galbo et al., 2021; Chen et al., 2022; Obradovic et al., 2022; Mou et al., 2023). In colorectal cancer, increased abundance of only the IL1R1+ iCAF subgroup, but not all other iCAFs, correlates with a lower overall survival rate (Koncina et al., 2023). Conversely, in pancreatic ductal adenocarcinoma, a higher iCAF abundance is linked to better prognosis and improved response to immunotherapy (Hu et al., 2022). These contradictory observations suggest that the presence of iCAFs in tumors and their impact on tumor behavior vary depending on the specific tumor type.

### Contribution of iCAFs to the development of chemoresistance

**FIGURE 5:**
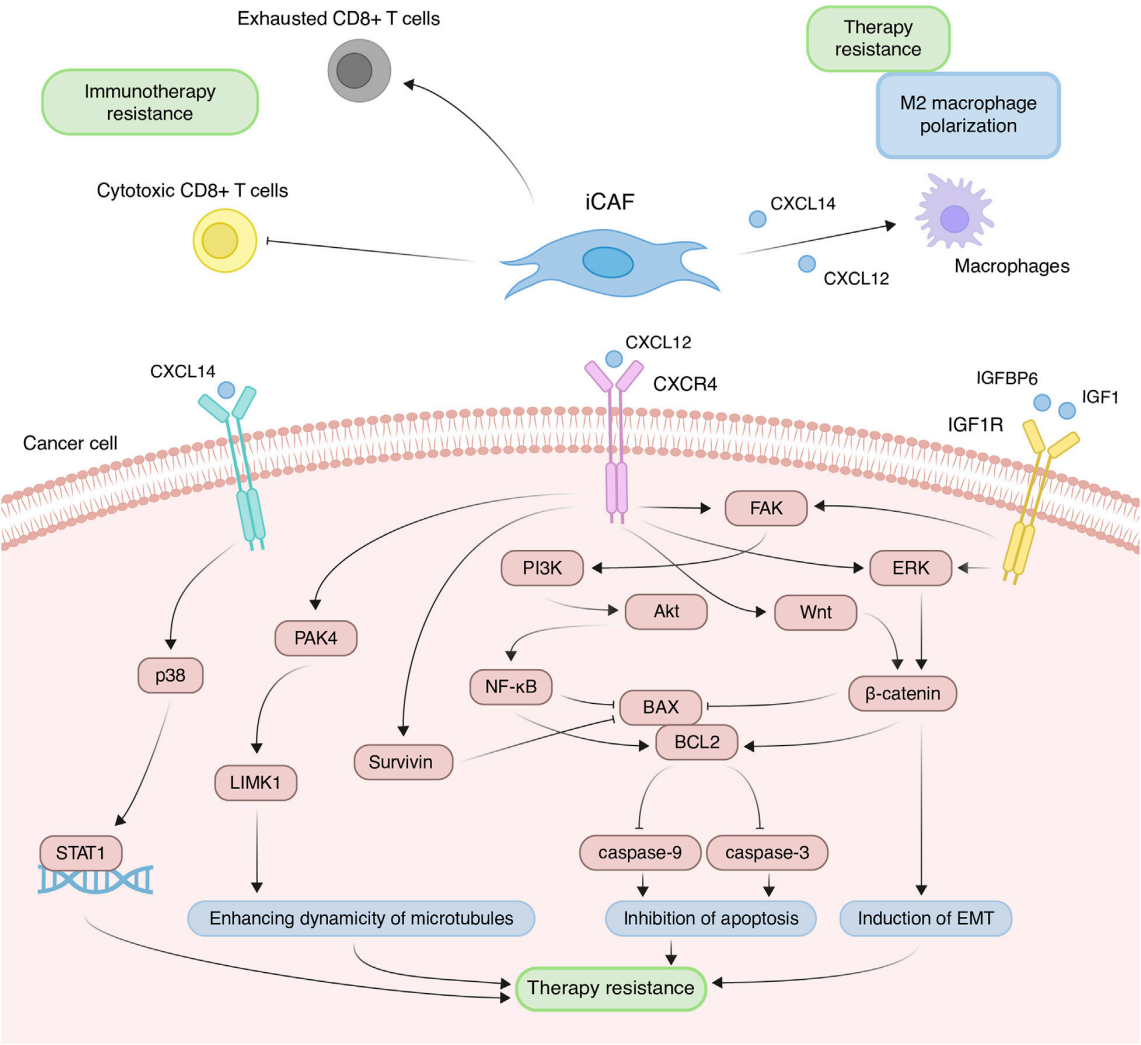
Principal roles and related mechanisms of inflammatory cancer-associated fibroblasts (iCAFs) in the development of therapeutic resistance of tumors. EMT, epithelial to mesenchymal transition.

Due to increased expression of inflammatory cytokines, iCAFs induce the differentiation of immune cells into pro-tumor populations and reduce the activity of immunostimulating cells, collectively contributing to the establishment of immunosuppressive TME. Across various tumor types, scRNA-seq studies consistently demonstrate that iCAFs contribute to the differentiation of macrophages towards an alternatively activated M2-like phenotype (Chen et al., 2020; Chen et al., 2022; Ma et al., 2023; Werba et al., 2023). This phenomenon is supported by the fact that cytokines typically produced by iCAFs play a pivotal role in macrophage polarization. For instance, in prostate, non-small cell lung, and colorectal cancers, CAF-derived CXCL12 and CXCL14 facilitate the M2 polarization of macrophages (Figure 5) (Augsten et al., 2014; Comito et al., 2014; Wu et al., 2022). M2 macrophages, distinct from classically activated (M1) ones, promote tumor proliferation, invasion, and immune suppression. Furthermore, M2-like macrophages can confer therapeutic tolerance to various anticancer drugs, including cisplatin, gemcitabine, tamoxifen, and doxorubicin (Wang et al., 2024). For example, CAF-derived CXCL12 accelerates colorectal cancer progression and induces cisplatin resistance *in vivo* by promoting the M2 polarization of macrophages (Figure 5) (Jiang et al., 2023).

In scRNA-seq studies, direct interactions between iCAFs and T cells have also been elucidated. iCAFs are presumed to interact with various tumor-infiltrating T cell populations through chemokine ligand-receptor interactions (Wu et al., 2020; Pu et al., 2021; Li et al., 2022). In triple-negative breast cancer, CXCL12+ iCAFs induce CD8^+^ T cell dysfunction (Figure 5) (Wu et al., 2020). Notably, the iCAFs signature exhibits a significant positive correlation with the exhausted CD8^+^ T cell signature but a negative correlation with cytotoxic CD8^+^ T cells in head and neck squamous cell carcinoma (Figure 5) (Mou et al., 2023). Cytotoxic CD8^+^ T cells are recognized as the most potent effectors in the anticancer immune response, forming the basis of current successful cancer immunotherapies (Raskov et al., 2021). Conversely, dysfunction and exhaustion of CD8^+^ T cells diminish the potential for sustained responses to checkpoint blockade (McGoverne et al., 2020).

In addition to modulating the tumor immune microenvironment, iCAFs exert direct effects on cancer cells, promoting resistance to anti-cancer therapy. Co-cultivation experiments involving ovarian, colorectal, and pancreatic cancer cells with CAFs or CAF supernatants have demonstrated a reduction in cisplatin- and gemcitabine-induced apoptosis, attributed to CAF-derived CXCL12 (Zhang et al., 2015; Morimoto et al., 2016; Wei et al., 2018; Zhang et al., 2020; Jiang et al., 2023). CXCL12-induced resistance primarily occurs via activation of the CXCL12/CXCR4 axis, initiating downstream signaling cascades in tumor cells, including Akt, ERK, and Wnt/β-catenin pathways (Figure 5) (Singh et al., 2010; Zhang et al., 2015; Zhang et al., 2020). These pathways regulate the expression of mitochondrial apoptotic proteins, such as BCL-2 and BAX. Reduced BAX/BCL-2 ratio in tumor cells leads to the inhibition of downstream caspase 3, which drives the terminal events of apoptosis. In colorectal cancer, CXCL12/CXCR4-mediated upregulation of miR-125b initiates a similar cascade to evade the effects of 5-fluorouracil (Yu et al., 2017). It is worth noting that a comparable mechanism of resistance to the cytotoxic effects of chemotherapeutic agents was observed in the interaction between myCAF-specific proteins and tumor cells. Suppressing effector caspases (caspase-3 and 9) may also occur upon activation of the CXCL12/CXCR4/survivin axis, resulting in decreased apoptosis and enhanced radiotherapy resistance in colon cancer cells (Figure 5) (Wang et al., 2017). Furthermore, the IL-6 autocrine loop, facilitated by Akt and ERK signaling pathway activation, contributes to CXCL12-induced gemcitabine chemoresistance in pancreatic cancer cells (Zhang et al., 2015). Additionally, CXCL12/CXCR4 signaling antagonizes docetaxel-induced G2/M phase cell cycle arrest by activating PAK4/LIMK1, enhancing microtubule dynamics (Figure 5). Consequently, this impedes docetaxel-mediated microtubule stabilization, suppresses its anti-mitotic effect, and promotes tumor cell survival (Bhardwaj et al., 2014). Collectively, these findings underscore the role of the CXCL12 axis in mediating cancer chemoresistance.

Less explored in the context of anticancer drug resistance, other iCAF markers warrant further investigation (Table 2). It is known that CAF-derived CXCL14 activates p38/STAT1 signaling in breast cancer cells, reducing paclitaxel-induced apoptosis (Figure 5) (Liu et al., 2017). Insulin-like growth factor IGF1 and insulin-like growth factor binding protein IGFBP6 exert contrasting effects on tumor cells, with pro-tumor and anti-tumor effects, respectively (Figure 5). Depending on the balance between secreted IGF and IGFBP proteins, CAFs can induce both osimertinib resistance and osimertinib sensitivity in lung cancer cells (Remsing Rix et al., 2022). Regarding complement system genes C3 and C7, there exists only indirect evidence suggesting their potential impact on anticancer therapy resistance. Elevated C7 expression predicts an unfavorable prognosis in patients treated with taxane-anthracycline chemotherapy (Zhang et al., 2021), while C3 expression is associated with resistance to 5-fluorouracil- and oxaliplatin-based chemotherapy in colorectal cancer (He et al., 2021). Consequently, the precise role of the iCAFs specific signature in tumor cell resistance to therapeutic agents remains incompletely characterized. A comprehensive investigation of iCAF markers could elucidate the contribution of this population to tumor resistance development.

**TABLE 2:** The role of inflammatory cancer-associated fibroblast markers in the development of therapeutic resistance of tumors.

Gene	Tumor type	Therapy	Effect/Mechanism	References
CXCL12	pancreatic cancer (*in vitro*, SW1990 и PANC-1)	gemcitabine	Cultivation of cancer cells with CAF-CM leads to increased resistance/Activation of SATB-1 expression	Wei et al. (2018)
CXCL12	pancreatic cancer (*in vitro*, MIA PaCa-2 and AsPC-1)	gemcitabine	Cultivation of cancer cells with CAFs leads to increased resistance	Morimoto et al. (2016)
CXCL12	pancreatic cancer (*in vitro*, Panc-1)	gemcitabine	Cultivation of cancer cells with CAF-CM leads to increased resistance/Activation of the FAK/Akt and ERK pathways leads to a subsequent increase in IL6 expression	Zhang et al. (2015)
CXCL12	pancreatic cancer (*in vitro*, Panc1 and MiaPaCa)	gemcitabine	Cultivation of cancer cells with CXCL12 leads to increased resistance/Activation of the FAK, Akt, and ERK pathways leads to a subsequent decrease in the BAX/BCL-2 ratio and the inhibition of downstream caspase 3	Singh et al. (2010)
CXCL12	ovarian cancer (*in vitro*, SKOV3)	cisplatin	Cultivation of cancer cells with CXCL12 leads to increased resistance	Dai et al. (2023)
CXCL12	ovarian cancer (*in vitro*, SKOV3 and OVCAR3)	cisplatin	Cultivation of cancer cells with CAF supernatants leads to increased resistance/Activation of the Wnt/β-catenin pathway	Zhang et al. (2020b)
CXCL12	colorectal cancer (*in vitro*, HCT116 and THP-1 with CAFs)	cisplatin	Cultivation of cancer cells with CAFs-derived CXCL12 leads to increased resistance/Induction of the M2 macrophages polarization	Jiang et al. (2023)
CXCL12	colorectal cancer (*in vitro*, HCT116 and SW620)	5-fluorouracil	Cultivation of cancer cells with CXCL12 leads to increased resistance/Activation of miR-125b expression leads to a subsequent decrease in the BAX/BCL-2 ratio and the inhibition of downstream caspase 3	Yu et al. (2017)
CXCL12	colon cancer (*in vitro*, HCT116)	radiotherapy	Cultivation of cancer cells with CXCL12 leads to increased resistance/Activation of survivin expression leads to a subsequent decrease in the BAX/BCL-2 ratio and the inhibition of downstream caspase 3 and caspase 9	Wang et al. (2017)
C3	colorectal cancer (patient tumor tissues)	FOLFOX	High-level of C3 expression is associated with a worse therapy response	He et al. (2021)
C3	colon cancer (mice tumor xenograft)	anti–PD-L1 treatment	C3 knockdown in cancer cells leads to decreased resistance/Deactivation of the C3a/C3aR/PI3Kγ pathway in macrophages	Zha et al. (2019)
C7	breast cancer (patient tumor tissues)	taxane-anthracycline	High-level of C7 expression is associated with a worse therapy response	Zhang et al. (2021c)
CXCL14	breast cancer (MCF7 and SKBR3)	paclitaxel	Cultivation of cancer cells with CAFs-derived CXCL14 leads to increased resistance/Activation the p38/STAT1 pathway	Liu et al. (2017a)
IGF-1	breast cancer (*in vitro*, MCF-7)	doxorubicin	Cultivation of cancer cells with IGF-1 leads to increased resistance	Gooch et al. (1999)
IGF-1	non-small cell lung cancer (*in vitro*, PC9GR)	osimertinib	Cultivation of cancer cells with IGF-1 leads to increased resistance	Remsing Rix et al. (2022)
IGFBP6	non-small cell lung cancer (*in vitro*, PC9GR)	osimertinib	Cultivation of cancer cells with IGFBP6 leads to decreased resistance	Remsing Rix et al. (2022)
CXCL12	prostate cancer (*in vitro*, C4-2 and PC3)	docetaxel	Cultivation of cancer cells with CXCL12 leads to increased resistance/Activation of the PAK4/LIMK1 pathway	Bhardwaj et al. (2014)
CXCL12	cervical cancer (*in vitro*, HeLa)	radiotherapy	Knockdown of CXCL12 in cancer cells leads to decreased resistance	Fu et al. (2018)

## Antigen-presenting subtype of cancer-associated fibroblasts—apCAFs

One of the pivotal mechanisms enabling tumors to evade immune surveillance involves attenuating the initiation of anti-tumor T-cell responses mediated by antigen-presenting cells (APCs). Within the TME, APCs, predominantly constituted by “professional” APCs including dendritic cells (DCs), macrophages, and B cells, orchestrate the activation of anti-cancer immunity (Wilke et al., 2011; Martin et al., 2015). Nonetheless, the initiation and regulation of anti-cancer immunity may also involve “non-professional” APCs, such as innate lymphoid cells, epithelial cells, endothelial cells, mast cells, granulocytes or fibroblasts (Kambayashi and Laufer, 2014; Park et al., 2022). Identified through scRNA-seq analysis of pancreatic tumors from mice, apCAFs represent a transcriptionally and functionally distinct CAF subtype, characterized by heightened expression of genes associated with MHC class II (MHC II)-mediated antigen presentation (Elyada et al., 2019). Subsequently, the presence of a separate apCAF population was confirmed by scRNA-seq data across more than 10 different types of human tumors (Zhang et al., 2020; Kieffer et al., 2020; Wang et al., 2021; Hu et al., 2022; Luo et al., 2022; Liu et al., 2023; Cords et al., 2023). The key genes that differentiated apCAFs from other tumor fibroblast subtypes in these studies include *HLA-DRA* (encoding MHC II alpha chain), *HLA-DRB1* (encoding MHC II beta chain) and *CD74* (encoding MHC II invariant chain). These genes underscore the involvement of apCAFs in critical biological processes such as antigen processing and presentation, allograft rejection, and inflammatory response.

While *ex vivo* experiments validated the capacity of apCAFs to present model antigens to CD4^+^ T cells (Elyada et al., 2019), their precise role within the TME remains ambiguous and, to a large extent, contradictory. “Professional” APCs and other MHC II-expressing tumor cells contribute to the initiation and maintenance of anti-tumor immunity through interaction with tumor-specific CD4^+^ T cells (Harryvan et al., 2021; Tay et al., 2021). To provoke the activation, proliferation and differentiation of T cells, APCs must present two signal molecule groups on their surface: MHC II molecules and costimulatory molecules like CD80, CD86, and CD40 (Liao et al., 2019). In turn, apCAFs, like other subtypes of CAFs, exhibit low expression of costimulatory molecules, hindering the full activation of T cells (Elyada et al., 2019; Friedman et al., 2020). However, in pancreatic cancer, apCAFs originating from the mesothelium have been implicated in inducing the transformation of naïve CD4^+^ T cells into FOXP3+ regulatory T cells in an antigen-specific manner, thereby fostering immune evasion within the tumor milieu (Huang et al., 2022). Conversely, in human lung non-small cell carcinomas, apCAFs have displayed tumor-suppressive properties (Kerdidani et al., 2022). apCAFs have been shown to directly activate effector CD4^+^ T cells infiltrating tumors through MHC II-mediated T-cell receptor stimulation. Additionally, the interaction between the ligand C1q, secreted by apCAFs, and the C1qbp receptor on the surface of effector CD4^+^ T cells confers protection of the latter against apoptosis. Conversely, targeted deletion of MHCII or C1q in fibroblasts markedly accelerates tumor growth.

scRNA-seq analysis has revealed a substantial upregulation of genes associated with the response to IFNγ in the apCAFs population (Zhang et al., 2020; Kieffer et al., 2020; Cords et al., 2023). Hence, the impact of IFNγ, purportedly secreted by T helper 1 cells, cytotoxic T lymphocytes, and natural killer cells (Gocher et al., 2022), likely stimulates the induction of the apCAF phenotype in tumors. Notably, apCAFs are predominantly detected in tumors expressing IFNγ. This assertion finds support in experiments where the injection of lung cancer cells lacking IFNγ/IFNγR into mice resulted in a significant downregulation of MHC II expression in CAFs (Kerdidani et al., 2022). Additionally, in pancreatic cancer, mesothelial cells have been identified as a source of apCAFs, undergoing a transition activated by signaling cascades involving IL-1 and TGFβ (Huang et al., 2022). scRNA-seq analysis suggests that the apCAFs population may represent a transitional state between tumor-associated macrophages and myofibroblasts (Luo et al., 2022).

In conclusion, the existence of a distinct subset of antigen-presenting CAFs, characterized by heightened expression of MHC II-associated genes within the tumor stromal microenvironment, is well-established. However, further elucidation of the conditions governing their activation and functional significance is imperative.

## Other subtypes of cancer-associated fibroblasts

In addition to the three primary populations of CAFs, scRNA-seq studies have unveiled several other subgroups (Figure 1). For instance, among the datasets, a distinct cluster of proliferative CAFs (pCAFs) or dividing CAFs (dCAFs) has been identified (Galbo et al., 2021; Li et al., 2022; Cords et al., 2023; Ma et al., 2023). These cells exhibit unique expression patterns of cell cycle-related genes (*TOP2A*, *TUBA1B*, and *MKI67*). Another cluster, termed interferon-response CAFs (ifnCAFs), is characterized by elevated expression of a set of IFNα and IFNγ response genes (*IFIT1*, *IFIT2*, *IFIT3*, *CXCL11*, and others) (Kieffer et al., 2020; Cords et al., 2023). Imaging Mass Cytometry analysis has shown that ifnCAFs are localized in close proximity to tumor cells (Cords et al., 2023). *In vitro* models have demonstrated that ifnCAFs exert a proliferative effect on MCF-7 breast cancer cells (Hosein et al., 2015). Conversely, the impact of effector CD8^+^ T cell-derived IFNγ on CAFs from ovarian tumors stimulates the expression of *GGT5* and leads to a reduction in glutathione levels in the fibroblast medium. Consequently, CAFs lose their ability to protect tumor cells from the cytotoxic effects of cisplatin, thereby enhancing therapeutic effectiveness (Wang et al., 2016). In a pan-cancer study, a cluster of metabolic CAFs (meCAFs) has been identified, associated with the response to hypoxia, glycolysis, and alanine, aspartate, and glutamate metabolism (Ma et al., 2023). Patients with pancreatic ductal adenocarcinoma exhibiting a high abundance of meCAFs faced an elevated risk of metastasis and an unfavorable prognosis; nevertheless, they demonstrated a remarkably improved response to immunotherapy (Wang et al., 2021). During scRNA-seq analysis of intrahepatic cholangiocarcinoma, two additional unique clusters of CAFs were identified: EMT-like CAFs, expressing high levels of epithelium-specific genes (*KRT19*, *KRT8*, and *SAA1*), and lipofibroblasts with increased expression of lipid metabolism genes (*APOA2*, *FABP1*, *FABP4*, and *FRZB*) (Zhang et al., 2020). In breast tumors, two additional rare populations have been identified: tumor-like CAFs (tCAFs) and reticular-like CAFs (rCAFs) (Cords et al., 2023). The first population is characterized by elevated expression of proliferation, migration, and metastasis-associated genes (*PDPN*, *MME*, *TMEM158*, and *NDRG1*), while markers of the second population coincide with those of reticular fibroblasts in lymphoid tissues (*CCL21* and *CCL19*).

Thus, the classification of CAFs remains complex, and the exact number of distinct CAF populations is still unknown. Currently, a few rare CAF populations can be identified using scRNA-seq methods on *ex vivo* data in certain tumor types. Discrepancies in research findings may be attributed to the existence of unique populations within specific tumor types or even among patients. Furthermore, fibroblast populations can transition from one to another, for example, from iCAFs to myCAFs (Öhlund et al., 2017), altering the number and representation of CAF populations throughout tumor progression. Challenges in identifying CAF populations in studies may also stem from the specificities of scRNA-seq analysis. For instance, some rare populations may go undetected due to the limited amount of analyzed material. Additionally, scRNA-seq analysis allows for the segmentation of all cells into an arbitrarily large number of clusters. Consequently, it is challenging to determine whether the identified clusters correspond to unique, rare CAF phenotypes or are solely related to analysis features and inaccuracies.

## Discussion

Despite the long-standing history of researching CAFs, the precise classification of individual populations remains a formidable challenge in the scientific community. While scRNA-seq technologies aid in unraveling the heterogeneity of CAFs, several unresolved issues persist. Presently, the existence of three primary CAF populations is firmly established: myCAFs, iCAFs, and apCAFs. While the characteristics of iCAFs and apCAFs demonstrate consistency across various studies, notable discrepancies emerge concerning myCAFs. Depending on the study, the definition of myCAFs encompasses two cellular populations with completely different transcriptional profiles. The distinctive features of one of these populations (RGS5+MYH11+ cells) overlap with the characteristic traits of pericytes. We propose a systematic approach for CAF classification: Firstly, during the analysis of RGS5+MYH11+ cells, it is imperative to confirm their fibroblastic nature and exclude pericytic identity. If confirmed as fibroblasts, we advocate referring to them as vCAFs, aligning with prior studies that distinguish pericytes from RGS5+MYH11+ CAFs (vCAFs) (Cords et al., 2023). On the other hand, myCAFs can be defined as fibroblasts actively expressing ECM genes. Such a classification strategy serves to prevent misinterpretation and ensures accurate characterization, particularly regarding pericyte misidentification.

In addition to the three primary populations, scRNA-seq technologies offer the capability to identify rare and low-abundance CAF populations across diverse tumor types (Zhang et al., 2020; Galbo et al., 2021; Li et al., 2022; Cords et al., 2023; Ma et al., 2023). Achieving a comprehensive and accurate classification of all conceivable CAF populations necessitates the confirmation of their existence *in vivo*. Moreover, it is imperative to ascertain whether these populations are universally present across tumor types but have remained undetected due to their scarcity, or if they are exclusive to specific tumor types. A thorough characterization of all CAF populations entails investigating activation mechanisms, reciprocal transitions between distinct subpopulations, and their spatial distribution within the TME. Our understanding of how these functional subsets evolve as tumors progress and metastasize to distant sites remains incomplete. It is important to understand that as the tumor advances, the abundance of certain CAF populations and their contributions to carcinogenesis may undergo dynamic changes. Furthermore, specific rare CAF populations identified through scRNA-seq analysis may directly correlate with tumor stage. Given that each CAF population plays a distinct role in cancer development, addressing these complexities holds promise for driving significant advancements in tumor diagnosis, treatment, and monitoring methodologies.

Given the role of CAFs in conferring resistance to chemotherapy through ECM remodeling, tumor cell proliferation promotion, and immune response suppression via secreted molecules, novel treatment avenues emerge. Therapeutic strategies targeting CAFs can be categorized into three main approaches: 1) development of agents directly targeting CAF surface markers (primarily ACTA2 and FAP); 2) inhibition of signaling pathways implicated in CAF activation (such as TGFβ); 3) targeting CAF-derived ECM proteins (Zhang et al., 2023). However, many preclinical studies have not observed significant anti-tumor effects or notable improvements in patient survival (Zhang et al., 2023). The clinical shortcomings of CAF-targeted therapy may stem from several factors. Firstly, the toxicity of current drugs largely results from the non-specificity of target existing proteins. Notably, CAF surface markers ACTA2 and FAP are expressed by other cells within the tumor microenvironment, undermining the precision of CAF-based therapeutic strategies (Kazakova et al., 2022). Additionally, not all CAFs express ACTA2 and FAP, further complicating the efficacy of CAF-targeted therapy. Secondly, the majority of ongoing preclinical trials focus on the entire CAF population, disregarding their heterogeneous nature and multifaceted functions within tumors (Zhang et al., 2023). As distinct CAF populations may exhibit both tumor-promoting and tumor-suppressive properties, targeting specific pro-tumoral CAF populations proves challenging. Reprogramming the phenotype of CAFs could potentially offer a more effective strategy. Nonetheless, the development of such therapeutic agents necessitates a clear understanding of which CAF populations in tumors exclusively possess tumor-suppressive properties. When devising clinically effective anti-CAF therapy with minimal systemic side effects, it is crucial to acknowledge the unique cellular composition of individual tumors, characterized by specific ratios of CAF populations. We anticipate that novel therapeutic approaches will emerge by reprogramming pro-tumor CAFs into an anti-tumor CAF population. Implementing such agents as first-line treatments holds promise for enhancing the efficacy of conventional chemotherapeutic drugs.

## References

[B1] Amrutkar M. Aasrum M. Verbeke C. S. Gladhaug I. P. (2019). Secretion of fibronectin by human pancreatic stellate cells promotes chemoresistance to gemcitabine in pancreatic cancer cells. BMC Cancer 19, 596. 10.1186/s12885-019-5803-1 31208372 PMC6580453

[B2] Assidicky R. Tokat U. M. Tarman I. O. Saatci O. Ersan P. G. Raza U. (2022). Targeting HIF1-alpha/miR-326/ITGA5 axis potentiates chemotherapy response in triple-negative breast cancer. Breast Cancer Res. Treat. 193, 331–348. 10.1007/s10549-022-06569-5 35338412 PMC9389626

[B3] Augsten M. Sjöberg E. Frings O. Vorrink S. U. Frijhoff J. Olsson E. (2014). Cancer-associated fibroblasts expressing CXCL14 rely upon NOS1-derived nitric oxide signaling for their tumor-supporting properties. Cancer Res. 74, 2999–3010. 10.1158/0008-5472.CAN-13-2740 24710408

[B4] Baltes F. Pfeifer V. Silbermann K. Caspers J. Wantoch von Rekowski K. Schlesinger M. (2020). β1-Integrin binding to collagen type 1 transmits breast cancer cells into chemoresistance by activating ABC efflux transporters. Biochim. Biophys. Acta Mol. Cell Res. 1867, 118663. 10.1016/j.bbamcr.2020.118663 31987794

[B5] Bartoschek M. Oskolkov N. Bocci M. Lövrot J. Larsson C. Sommarin M. (2018). Spatially and functionally distinct subclasses of breast cancer-associated fibroblasts revealed by single cell RNA sequencing. Nat. Commun. 9, 5150. 10.1038/s41467-018-07582-3 30514914 PMC6279758

[B6] Bates A. L. Pickup M. W. Hallett M. A. Dozier E. A. Thomas S. Fingleton B. (2015). Stromal matrix metalloproteinase 2 regulates collagen expression and promotes the outgrowth of experimental metastases. J. Pathol. 235, 773–783. 10.1002/path.4493 25469981 PMC4357558

[B7] Bhardwaj A. Srivastava S. K. Singh S. Arora S. Tyagi N. Andrews J. (2014). CXCL12/CXCR4 signaling counteracts docetaxel-induced microtubule stabilization via p21-activated kinase 4-dependent activation of LIM domain kinase 1. Oncotarget 5, 11490–11500. 10.18632/oncotarget.2571 25359780 PMC4294337

[B8] Biffi G. Oni T. E. Spielman B. Hao Y. Elyada E. Park Y. (2019). IL1-Induced JAK/STAT signaling is antagonized by TGFβ to shape CAF heterogeneity in pancreatic ductal adenocarcinoma. Cancer Discov. 9, 282–301. 10.1158/2159-8290.CD-18-0710 30366930 PMC6368881

[B9] Bischoff P. Trinks A. Wiederspahn J. Obermayer B. Pett J. P. Jurmeister P. (2022). The single-cell transcriptional landscape of lung carcinoid tumors. Int. J. Cancer 150, 2058–2071. 10.1002/ijc.33995 35262195

[B10] Che L.-H. Liu J.-W. Huo J.-P. Luo R. Xu R.-M. He C. (2021). A single-cell atlas of liver metastases of colorectal cancer reveals reprogramming of the tumor microenvironment in response to preoperative chemotherapy. Cell Discov. 7, 80. 10.1038/s41421-021-00312-y 34489408 PMC8421363

[B11] Chen H. Yang W. Xue X. Li Y. Jin Z. Ji Z. (2022). Integrated analysis revealed an inflammatory cancer-associated fibroblast-based subtypes with promising implications in predicting the prognosis and immunotherapeutic response of bladder cancer patients. Int. J. Mol. Sci. 23, 15970. 10.3390/ijms232415970 36555612 PMC9781727

[B12] Chen K. Wang Q. Li M. Guo H. Liu W. Wang F. (2021). Single-cell RNA-seq reveals dynamic change in tumor microenvironment during pancreatic ductal adenocarcinoma malignant progression. EBioMedicine 66, 103315. 10.1016/j.ebiom.2021.103315 33819739 PMC8047497

[B13] Chen Z. Zhou L. Liu L. Hou Y. Xiong M. Yang Y. (2020). Single-cell RNA sequencing highlights the role of inflammatory cancer-associated fibroblasts in bladder urothelial carcinoma. Nat. Commun. 11, 5077. 10.1038/s41467-020-18916-5 33033240 PMC7545162

[B14] Cho C. Horzempa C. Jones D. McKeown-Longo P. J. (2016). The fibronectin III-1 domain activates a PI3-Kinase/Akt signaling pathway leading to αvβ5 integrin activation and TRAIL resistance in human lung cancer cells. BMC Cancer 16, 574. 10.1186/s12885-016-2621-6 27484721 PMC4970220

[B15] Chu L. Wang F. Zhang W. Li H.-F. Xu J. Tong X.-W. (2020). Periostin secreted by carcinoma-associated fibroblasts promotes ovarian cancer cell platinum resistance through the PI3K/Akt signaling pathway. Technol. Cancer Res. Treat. 19, 1533033820977535. 10.1177/1533033820977535 33302812 PMC7734496

[B16] Comito G. Giannoni E. Segura C. P. Barcellos-de-Souza P. Raspollini M. R. Baroni G. (2014). Cancer-associated fibroblasts and M2-polarized macrophages synergize during prostate carcinoma progression. Oncogene 33, 2423–2431. 10.1038/onc.2013.191 23728338

[B17] Cords L. Tietscher S. Anzeneder T. Langwieder C. Rees M. de Souza N. (2023). Cancer-associated fibroblast classification in single-cell and spatial proteomics data. Nat. Commun. 14, 4294. 10.1038/s41467-023-39762-1 37463917 PMC10354071

[B18] Cox T. R. Erler J. T. (2011). Remodeling and homeostasis of the extracellular matrix: implications for fibrotic diseases and cancer. Dis. Model Mech. 4, 165–178. 10.1242/dmm.004077 21324931 PMC3046088

[B19] Dai J.-M. Sun K. Li C. Cheng M. Guan J.-H. Yang L.-N. (2023). Cancer-associated fibroblasts contribute to cancer metastasis and apoptosis resistance in human ovarian cancer via paracrine SDF-1α. Clin. Transl. Oncol. 25, 1606–1616. 10.1007/s12094-022-03054-9 36593384

[B20] Dangi-Garimella S. Sahai V. Ebine K. Kumar K. Munshi H. G. (2013). Three-dimensional collagen I promotes gemcitabine resistance *in vitro* in pancreatic cancer cells through HMGA2-dependent histone acetyltransferase expression. PLoS One 8, e64566. 10.1371/journal.pone.0064566 23696899 PMC3655998

[B21] Deng M. Lin J. Nowsheen S. Liu T. Zhao Y. Villalta P. W. (2020). Extracellular matrix stiffness determines DNA repair efficiency and cellular sensitivity to genotoxic agents. Sci. Adv. 6, eabb2630. 10.1126/sciadv.abb2630 32917705 PMC7486107

[B22] Deng Y. Tan Y. Zhou D. Bai Y. Cao T. Zhong C. (2022). Single-cell RNA-sequencing atlas reveals the tumor microenvironment of metastatic high-grade serous ovarian carcinoma. Front. Immunol. 13, 923194. 10.3389/fimmu.2022.923194 35935940 PMC9354882

[B23] Dominguez C. X. Müller S. Keerthivasan S. Koeppen H. Hung J. Gierke S. (2020). Single-cell RNA sequencing reveals stromal evolution into LRRC15+ myofibroblasts as a determinant of patient response to cancer immunotherapy. Cancer Discov. 10, 232–253. 10.1158/2159-8290.CD-19-0644 31699795

[B24] Eke I. Storch K. Krause M. Cordes N. (2013). Cetuximab attenuates its cytotoxic and radiosensitizing potential by inducing fibronectin biosynthesis. Cancer Res. 73, 5869–5879. 10.1158/0008-5472.CAN-13-0344 23950208

[B25] Elango J. Hou C. Bao B. Wang S. Maté Sánchez de Val J. E. Wenhui W. (2022). The molecular interaction of collagen with cell receptors for biological function. Polymers 14, 876. 10.3390/polym14050876 35267698 PMC8912536

[B26] Elyada E. Bolisetty M. Laise P. Flynn W. F. Courtois E. T. Burkhart R. A. (2019). Cross-species single-cell analysis of pancreatic ductal adenocarcinoma reveals antigen-presenting cancer-associated fibroblasts. Cancer Discov. 9, 1102–1123. 10.1158/2159-8290.CD-19-0094 31197017 PMC6727976

[B27] Friedman G. Levi-Galibov O. David E. Bornstein C. Giladi A. Dadiani M. (2020). Cancer-associated fibroblast compositions change with breast cancer progression linking the ratio of S100A4^+^ and PDPN^+^ CAFs to clinical outcome. Nat. Cancer 1, 692–708. 10.1038/s43018-020-0082-y 35122040 PMC7617059

[B28] Fu Z. Zhang P. Luo H. Huang H. Wang F. (2018). CXCL12 modulates the radiosensitivity of cervical cancer by regulating CD44. Mol. Med. Rep. 18, 5101–5108. 10.3892/mmr.2018.9554 30320394

[B29] Fuyuhiro Y. Yashiro M. Noda S. Kashiwagi S. Matsuoka J. Doi Y. (2011). Upregulation of cancer-associated myofibroblasts by TGF-β from scirrhous gastric carcinoma cells. Br. J. Cancer 105, 996–1001. 10.1038/bjc.2011.330 21863023 PMC3185946

[B30] Galbo P. M. Zang X. Zheng D. (2021). Molecular features of cancer-associated fibroblast subtypes and their implication on cancer pathogenesis, prognosis, and immunotherapy resistance. Clin. Cancer Res. 27, 2636–2647. 10.1158/1078-0432.CCR-20-4226 33622705 PMC8102353

[B31] Gao W. Liu Y. Qin R. Liu D. Feng Q. (2016). Silence of fibronectin 1 increases cisplatin sensitivity of non-small cell lung cancer cell line. Biochem. Biophys. Res. Commun. 476, 35–41. 10.1016/j.bbrc.2016.05.081 27207836

[B32] Ghosh S. Albitar L. LeBaron R. Welch W. R. Samimi G. Birrer M. J. (2010). Up-regulation of stromal versican expression in advanced stage serous ovarian cancer. Gynecol. Oncol. 119, 114–120. 10.1016/j.ygyno.2010.05.029 20619446 PMC3000175

[B33] Giguelay A. Turtoi E. Khelaf L. Tosato G. Dadi I. Chastel T. (2022). The landscape of cancer-associated fibroblasts in colorectal cancer liver metastases. Theranostics 12, 7624–7639. 10.7150/thno.72853 36438498 PMC9691344

[B34] Gocher A. M. Workman C. J. Vignali D. A. A. (2022). Interferon-γ: teammate or opponent in the tumour microenvironment? Nat. Rev. Immunol. 22, 158–172. 10.1038/s41577-021-00566-3 34155388 PMC8688586

[B35] Gooch J. L. Van Den Berg C. L. Yee D. (1999). Insulin-like growth factor (IGF)-I rescues breast cancer cells from chemotherapy-induced cell death--proliferative and anti-apoptotic effects. Breast Cancer Res. Treat. 56, 1–10. 10.1023/a:1006208721167 10517338

[B36] Guo Y. Zhai J. Zhang J. Ni C. Zhou H. (2019). Improved radiotherapy sensitivity of nasopharyngeal carcinoma cells by miR-29-3p targeting COL1A1 3’-utr. Med. Sci. Monit. 25, 3161–3169. 10.12659/MSM.915624 31034464 PMC6503752

[B37] Harryvan T. J. de Lange S. Hawinkels LJAC Verdegaal E. M. E. (2021). The ABCs of antigen presentation by stromal non-professional antigen-presenting cells. Int. J. Mol. Sci. 23, 137. 10.3390/ijms23010137 35008560 PMC8745042

[B38] He X.-S. Zou S.-Y. Yao J.-L. Yu W. Deng Z.-Y. Wang J.-R. (2021). Transcriptomic analysis identifies complement component 3 as a potential predictive biomarker for chemotherapy resistance in colorectal cancer. Front. Mol. Biosci. 8, 763652. 10.3389/fmolb.2021.763652 34722636 PMC8554154

[B39] Hornburg M. Desbois M. Lu S. Guan Y. Lo A. A. Kaufman S. (2021). Single-cell dissection of cellular components and interactions shaping the tumor immune phenotypes in ovarian cancer. Cancer Cell 39, 928–944.e6. e6. 10.1016/j.ccell.2021.04.004 33961783

[B40] Hosein A. N. Livingstone J. Buchanan M. Reid J. F. Hallett M. Basik M. (2015). A functional *in vitro* model of heterotypic interactions reveals a role for interferon-positive carcinoma associated fibroblasts in breast cancer. BMC Cancer 15, 130. 10.1186/s12885-015-1117-0 25884794 PMC4369836

[B41] Hu B. Wu C. Mao H. Gu H. Dong H. Yan J. (2022). Subpopulations of cancer-associated fibroblasts link the prognosis and metabolic features of pancreatic ductal adenocarcinoma. Ann. Transl. Med. 10, 262. 10.21037/atm-22-407 35402584 PMC8987890

[B42] Hu W. Jin P. Liu W. (2016). Periostin contributes to cisplatin resistance in human non-small cell lung cancer A549 cells via activation of Stat3 and Akt and upregulation of survivin. Cell Physiol. Biochem. 38, 1199–1208. 10.1159/000443068 26982182

[B43] Huang H. Wang Z. Zhang Y. Pradhan R. N. Ganguly D. Chandra R. (2022). Mesothelial cell-derived antigen-presenting cancer-associated fibroblasts induce expansion of regulatory T cells in pancreatic cancer. Cancer Cell 40, 656–673.e7. 10.1016/j.ccell.2022.04.011 35523176 PMC9197998

[B44] Huang M. Fu M. Wang J. Xia C. Zhang H. Xiong Y. (2021). TGF-β1-activated cancer-associated fibroblasts promote breast cancer invasion, metastasis and epithelial-mesenchymal transition by autophagy or overexpression of FAP-α. Biochem. Pharmacol. 188, 114527. 10.1016/j.bcp.2021.114527 33741330

[B45] Jiang H. Ge H. Shi Y. Yuan F. Yue H. (2023). CAFs secrete CXCL12 to accelerate the progression and cisplatin resistance of colorectal cancer through promoting M2 polarization of macrophages. Med. Oncol. 40, 90. 10.1007/s12032-023-01953-7 36737590

[B46] Joyce M. H. Lu C. James E. R. Hegab R. Allen S. C. Suggs L. J. (2018). Phenotypic basis for matrix stiffness-dependent chemoresistance of breast cancer cells to doxorubicin. Front. Oncol. 8, 337. 10.3389/fonc.2018.00337 30234012 PMC6134055

[B47] Kambayashi T. Laufer T. M. (2014). Atypical MHC class II-expressing antigen-presenting cells: can anything replace a dendritic cell? Nat. Rev. Immunol. 14, 719–730. 10.1038/nri3754 25324123

[B48] Kazakova A. N. Anufrieva K. S. Ivanova O. M. Shnaider P. V. Malyants I. K. Aleshikova O. I. (2022). Deeper insights into transcriptional features of cancer-associated fibroblasts: an integrated meta-analysis of single-cell and bulk RNA-sequencing data. Front. Cell Dev. Biol. 10, 825014. 10.3389/fcell.2022.825014 36263012 PMC9574913

[B49] Kerdidani D. Aerakis E. Verrou K.-M. Angelidis I. Douka K. Maniou M.-A. (2022). Lung tumor MHCII immunity depends on *in situ* antigen presentation by fibroblasts. J. Exp. Med. 219, e20210815. 10.1084/jem.20210815 35029648 PMC8764966

[B50] Khera N. Rajkumar A. S. Abdulkader M. Alkurdi K. Liu Z. Ma H. (2023). Identification of multidrug chemoresistant genes in head and neck squamous cell carcinoma cells. Mol. Cancer 22, 146. 10.1186/s12943-023-01846-3 37667354 PMC10476423

[B51] Kieffer Y. Hocine H. R. Gentric G. Pelon F. Bernard C. Bourachot B. (2020). Single-cell analysis reveals fibroblast clusters linked to immunotherapy resistance in cancer. Cancer Discov. 10, 1330–1351. 10.1158/2159-8290.CD-19-1384 32434947

[B52] Koncina E. Nurmik M. Pozdeev V. I. Gilson C. Tsenkova M. Begaj R. (2023). IL1R1+ cancer-associated fibroblasts drive tumor development and immunosuppression in colorectal cancer. Nat. Commun. 14, 4251. 10.1038/s41467-023-39953-w 37460545 PMC10352362

[B53] Kun-Peng Z. Chun-Lin Z. Xiao-Long M. Lei Z. (2019). Fibronectin-1 modulated by the long noncoding RNA OIP5-AS1/miR-200b-3p axis contributes to doxorubicin resistance of osteosarcoma cells. J. Cell Physiol. 234, 6927–6939. 10.1002/jcp.27435 30204936

[B54] Lambrechts D. Wauters E. Boeckx B. Aibar S. Nittner D. Burton O. (2018). Phenotype molding of stromal cells in the lung tumor microenvironment. Nat. Med. 24, 1277–1289. 10.1038/s41591-018-0096-5 29988129

[B55] Li C. Wu H. Guo L. Liu D. Yang S. Li S. (2022a). Single-cell transcriptomics reveals cellular heterogeneity and molecular stratification of cervical cancer. Commun. Biol. 5, 1208. 10.1038/s42003-022-04142-w 36357663 PMC9649750

[B56] Li M. Ding W. Wang Y. Ma Y. Du F. (2023). Development and validation of a gene signature for pancreatic cancer: based on inflammatory response-related genes. Environ. Sci. Pollut. Res. Int. 30, 17166–17178. 10.1007/s11356-022-23252-w 36192587

[B57] Li X. Sun Z. Peng G. Xiao Y. Guo J. Wu B. (2022c). Single-cell RNA sequencing reveals a pro-invasive cancer-associated fibroblast subgroup associated with poor clinical outcomes in patients with gastric cancer. Theranostics 12, 620–638. 10.7150/thno.60540 34976204 PMC8692898

[B58] Li Y. Hu X. Lin R. Zhou G. Zhao L. Zhao D. (2022b). Single-cell landscape reveals active cell subtypes and their interaction in the tumor microenvironment of gastric cancer. Theranostics 12, 3818–3833. 10.7150/thno.71833 35664061 PMC9131288

[B59] Liao P. Wang H. Tang Y.-L. Tang Y.-J. Liang X.-H. (2019). The common costimulatory and coinhibitory signaling molecules in head and neck squamous cell carcinoma. Front. Immunol. 10, 2457. 10.3389/fimmu.2019.02457 31708918 PMC6819372

[B60] Lin W. Noel P. Borazanci E. H. Lee J. Amini A. Han I. W. (2020). Single-cell transcriptome analysis of tumor and stromal compartments of pancreatic ductal adenocarcinoma primary tumors and metastatic lesions. Genome Med. 12, 80. 10.1186/s13073-020-00776-9 32988401 PMC7523332

[B61] Liu C. Zhang M. Yan X. Ni Y. Gong Y. Wang C. (2023b). Single-cell dissection of cellular and molecular features underlying human cervical squamous cell carcinoma initiation and progression. Sci. Adv. 9, eadd8977. 10.1126/sciadv.add8977 36706185 PMC9882988

[B62] Liu M. Smith R. Liby T. Chiotti K. López C. S. Korkola J. E. (2022). INHBA is a mediator of aggressive tumor behavior in HER2+ basal breast cancer. Breast Cancer Res. 24, 18. 10.1186/s13058-022-01512-4 35248133 PMC8898494

[B63] Liu S. Liao G. Li G. (2017b). Regulatory effects of COL1A1 on apoptosis induced by radiation in cervical cancer cells. Cancer Cell Int. 17, 73. 10.1186/s12935-017-0443-5 28775672 PMC5534093

[B64] Liu T. Xia R. Li C. Chen X. Cai X. Li W. (2021). mRNA expression level of *CDH2*, *LEP*, *POSTN*, *TIMP1* and *VEGFC* modulates 5-fluorouracil resistance in colon cancer cells. Exp. Ther. Med. 22, 1023. 10.3892/etm.2021.10455 34373709 PMC8343572

[B65] Liu W. Wang M. Wang M. Liu M. (2023a). Single-cell and bulk RNA sequencing reveal cancer-associated fibroblast heterogeneity and a prognostic signature in prostate cancer. Medicine 102, e34611. 10.1097/MD.0000000000034611 37565899 PMC10419654

[B66] Liu Y. Li F. Gao F. Xing L. Qin P. Liang X. (2016). Periostin promotes the chemotherapy resistance to gemcitabine in pancreatic cancer. Tumour Biol. 37, 15283–15291. 10.1007/s13277-016-5321-6 27696296

[B67] Liu Y. Zhang J. Sun X. Su Q. You C. (2017a). Down-regulation of miR-29b in carcinoma associated fibroblasts promotes cell growth and metastasis of breast cancer. Oncotarget 8, 39559–39570. 10.18632/oncotarget.17136 28465475 PMC5503632

[B68] Loret N. Vandamme N. De Coninck J. Taminau J. De Clercq K. Blancke G. (2022). Distinct transcriptional programs in ascitic and solid cancer cells induce different responses to chemotherapy in high-grade serous ovarian cancer. Mol. Cancer Res. 20, 1532–1547. 10.1158/1541-7786.MCR-21-0565 35749080

[B69] Luo H. Xia X. Huang L.-B. An H. Cao M. Kim G. D. (2022). Pan-cancer single-cell analysis reveals the heterogeneity and plasticity of cancer-associated fibroblasts in the tumor microenvironment. Nat. Commun. 13, 6619. 10.1038/s41467-022-34395-2 36333338 PMC9636408

[B70] Ma C. Yang C. Peng A. Sun T. Ji X. Mi J. (2023). Pan-cancer spatially resolved single-cell analysis reveals the crosstalk between cancer-associated fibroblasts and tumor microenvironment. Mol. Cancer 22, 170. 10.1186/s12943-023-01876-x 37833788 PMC10571470

[B71] Mak T. K. Li X. Huang H. Wu K. Huang Z. He Y. (2022). The cancer-associated fibroblast-related signature predicts prognosis and indicates immune microenvironment infiltration in gastric cancer. Front. Immunol. 13, 951214. 10.3389/fimmu.2022.951214 35967313 PMC9372353

[B72] Martin K. Schreiner J. Zippelius A. (2015). Modulation of APC function and anti-tumor immunity by anti-cancer drugs. Front. Immunol. 6, 501. 10.3389/fimmu.2015.00501 26483791 PMC4586505

[B73] McGoverne I. Dunn J. Batham J. Tu W. J. Chrisp J. Rao S. (2020). Epitherapy and immune checkpoint blockade: using epigenetic reinvigoration of exhausted and dysfunctional T cells to reimburse immunotherapy response. BMC Immunol. 21, 22. 10.1186/s12865-020-00353-0 32316916 PMC7175524

[B74] Mhaidly R. Mechta-Grigoriou F. (2021). Role of cancer-associated fibroblast subpopulations in immune infiltration, as a new means of treatment in cancer. Immunol. Rev. 302, 259–272. 10.1111/imr.12978 34013544 PMC8360036

[B75] Min K.-W. Kim D.-H. Noh Y.-K. Son B. K. Kwon M. J. Moon J.-Y. (2021). Cancer-associated fibroblasts are associated with poor prognosis in solid type of lung adenocarcinoma in a machine learning analysis. Sci. Rep. 11, 16779. 10.1038/s41598-021-96344-1 34408230 PMC8373913

[B76] Morimoto M. Matsuo Y. Koide S. Tsuboi K. Shamoto T. Sato T. (2016). Enhancement of the CXCL12/CXCR4 axis due to acquisition of gemcitabine resistance in pancreatic cancer: effect of CXCR4 antagonists. BMC Cancer 16, 305. 10.1186/s12885-016-2340-z 27175473 PMC4866076

[B77] Mou T. Zhu H. Jiang Y. Xu X. Cai L. Zhong Y. (2023). Heterogeneity of cancer-associated fibroblasts in head and neck squamous cell carcinoma. Transl. Oncol. 35, 101717. 10.1016/j.tranon.2023.101717 37320872 PMC10277597

[B78] Muhl L. Genové G. Leptidis S. Liu J. He L. Mocci G. (2020). Single-cell analysis uncovers fibroblast heterogeneity and criteria for fibroblast and mural cell identification and discrimination. Nat. Commun. 11, 3953. 10.1038/s41467-020-17740-1 32769974 PMC7414220

[B79] Nallanthighal S. Rada M. Heiserman J. P. Cha J. Sage J. Zhou B. (2020). Inhibition of collagen XI alpha 1-induced fatty acid oxidation triggers apoptotic cell death in cisplatin-resistant ovarian cancer. Cell Death Dis. 11, 258. 10.1038/s41419-020-2442-z 32312965 PMC7171147

[B80] Obradovic A. Graves D. Korrer M. Wang Y. Roy S. Naveed A. (2022). Immunostimulatory cancer-associated fibroblast subpopulations can predict immunotherapy response in head and neck cancer. Clin. Cancer Res. 28, 2094–2109. 10.1158/1078-0432.CCR-21-3570 35262677 PMC9161438

[B81] Öhlund D. Handly-Santana A. Biffi G. Elyada E. Almeida A. S. Ponz-Sarvise M. (2017). Distinct populations of inflammatory fibroblasts and myofibroblasts in pancreatic cancer. J. Exp. Med. 214, 579–596. 10.1084/jem.20162024 28232471 PMC5339682

[B82] Olbrecht S. Busschaert P. Qian J. Vanderstichele A. Loverix L. Van Gorp T. (2021). High-grade serous tubo-ovarian cancer refined with single-cell RNA sequencing: specific cell subtypes influence survival and determine molecular subtype classification. Genome Med. 13, 111. 10.1186/s13073-021-00922-x 34238352 PMC8268616

[B83] Ouyang Q. Liu Y. Tan J. Li J. Yang D. Zeng F. (2019). Loss of ZNF587B and SULF1 contributed to cisplatin resistance in ovarian cancer cell lines based on Genome-scale CRISPR/Cas9 screening. Am. J. Cancer Res. 9, 988–998. 31218106 PMC6556596

[B84] Pan C.-W. Shen Z.-J. Wu T.-T. Tang X.-Y. Wang M. Sun J. (2009). Cell adhesion to fibronectin induces mitomycin C resistance in bladder cancer cells. BJU Int. 104, 1774–1779. 10.1111/j.1464-410X.2009.08639.x 19624598

[B85] Pan J. Ma Z. Liu B. Qian H. Shao X. Liu J. (2023). Identification of cancer-associated fibroblasts subtypes in prostate cancer. Front. Immunol. 14, 1133160. 10.3389/fimmu.2023.1133160 37033924 PMC10080037

[B86] Park J. Wang L. Ho P.-C. (2022). Metabolic guidance and stress in tumors modulate antigen-presenting cells. Oncogenesis 11, 62. 10.1038/s41389-022-00438-y 36244976 PMC9573874

[B87] Ping Q. Yan R. Cheng X. Wang W. Zhong Y. Hou Z. (2021). Cancer-associated fibroblasts: overview, progress, challenges, and directions. Cancer Gene Ther. 28, 984–999. 10.1038/s41417-021-00318-4 33712707

[B88] Pontiggia O. Sampayo R. Raffo D. Motter A. Xu R. Bissell M. J. (2012). The tumor microenvironment modulates tamoxifen resistance in breast cancer: a role for soluble stromal factors and fibronectin through β1 integrin. Breast Cancer Res. Treat. 133, 459–471. 10.1007/s10549-011-1766-x 21935603 PMC3719875

[B89] Pu W. Shi X. Yu P. Zhang M. Liu Z. Tan L. (2021). Single-cell transcriptomic analysis of the tumor ecosystems underlying initiation and progression of papillary thyroid carcinoma. Nat. Commun. 12, 6058. 10.1038/s41467-021-26343-3 34663816 PMC8523550

[B90] Qin P. Chen H. Wang Y. Huang L. Huang K. Xiao G. (2023b). Cancer-associated fibroblasts undergoing neoadjuvant chemotherapy suppress rectal cancer revealed by single-cell and spatial transcriptomics. Cell Rep. Med. 4, 101231. 10.1016/j.xcrm.2023.101231 37852187 PMC10591051

[B91] Qin Y. Zu X. Li Y. Han Y. Tan J. Cai C. (2023a). A cancer-associated fibroblast subtypes-based signature enables the evaluation of immunotherapy response and prognosis in bladder cancer. iScience 26, 107722. 10.1016/j.isci.2023.107722 37694141 PMC10485638

[B92] Rada M. Nallanthighal S. Cha J. Ryan K. Sage J. Eldred C. (2018). Inhibitor of apoptosis proteins (IAPs) mediate collagen type XI alpha 1-driven cisplatin resistance in ovarian cancer. Oncogene 37, 4809–4820. 10.1038/s41388-018-0297-x 29769618

[B93] Raskov H. Orhan A. Christensen J. P. Gögenur I. (2021). Cytotoxic CD8+ T cells in cancer and cancer immunotherapy. Br. J. Cancer 124, 359–367. 10.1038/s41416-020-01048-4 32929195 PMC7853123

[B94] Remsing Rix L. L. Sumi N. J. Hu Q. Desai B. Bryant A. T. Li X. (2022). IGF-binding proteins secreted by cancer-associated fibroblasts induce context-dependent drug sensitization of lung cancer cells. Sci. Signal 15, eabj5879. 10.1126/scisignal.abj5879 35973030 PMC9528501

[B95] Rice A. J. Cortes E. Lachowski D. Cheung B. C. H. Karim S. A. Morton J. P. (2017). Matrix stiffness induces epithelial-mesenchymal transition and promotes chemoresistance in pancreatic cancer cells. Oncogenesis 6, e352. 10.1038/oncsis.2017.54 28671675 PMC5541706

[B96] Sahai E. Astsaturov I. Cukierman E. DeNardo D. G. Egeblad M. Evans R. M. (2020). A framework for advancing our understanding of cancer-associated fibroblasts. Nat. Rev. Cancer 20, 174–186. 10.1038/s41568-019-0238-1 31980749 PMC7046529

[B97] Shen L. Yang M. Lin Q. Zhang Z. Zhu B. Miao C. (2016). COL11A1 is overexpressed in recurrent non-small cell lung cancer and promotes cell proliferation, migration, invasion and drug resistance. Oncol. Rep. 36, 877–885. 10.3892/or.2016.4869 27373316

[B98] Shen Y. Wang X. Lu J. Salfenmoser M. Wirsik N. M. Schleussner N. (2020). Reduction of liver metastasis stiffness improves response to bevacizumab in metastatic colorectal cancer. Cancer Cell 37, 800–817. 10.1016/j.ccell.2020.05.005 32516590

[B99] Singh S. Srivastava S. K. Bhardwaj A. Owen L. B. Singh A. P. (2010). CXCL12-CXCR4 signalling axis confers gemcitabine resistance to pancreatic cancer cells: a novel target for therapy. Br. J. Cancer 103, 1671–1679. 10.1038/sj.bjc.6605968 21045835 PMC2994230

[B100] Song J. Wei R. Huo S. Liu C. Liu X. (2022). Versican enrichment predicts poor prognosis and response to adjuvant therapy and immunotherapy in gastric cancer. Front. Immunol. 13, 960570. 10.3389/fimmu.2022.960570 36203562 PMC9530562

[B101] Song Y. Kim J.-S. Choi E. K. Kim J. Kim K. M. Seo H. R. (2017). TGF-β-independent CTGF induction regulates cell adhesion mediated drug resistance by increasing collagen I in HCC. Oncotarget 8, 21650–21662. 10.18632/oncotarget.15521 28423507 PMC5400613

[B102] Staub J. Chien J. Pan Y. Qian X. Narita K. Aletti G. (2007). Epigenetic silencing of HSulf-1 in ovarian cancer:implications in chemoresistance. Oncogene 26, 4969–4978. 10.1038/sj.onc.1210300 17310998

[B103] Stylianopoulos T. Munn L. L. Jain R. K. (2018). Reengineering the physical microenvironment of tumors to improve drug delivery and efficacy: from mathematical modeling to bench to bedside. Trends Cancer Res. 4, 292–319. 10.1016/j.trecan.2018.02.005 PMC5930008 29606314

[B104] Sugimoto H. Mundel T. M. Kieran M. W. Kalluri R. (2006). Identification of fibroblast heterogeneity in the tumor microenvironment. Cancer Biol. Ther. 5, 1640–1646. 10.4161/cbt.5.12.3354 17106243

[B105] Sun L. Guo S. Xie Y. Yao Y. (2023). The characteristics and the multiple functions of integrin β1 in human cancers. J. Transl. Med. 21, 787. 10.1186/s12967-023-04696-1 37932738 PMC10629185

[B106] Takatsu F. Suzawa K. Tomida S. Thu Y. M. Sakaguchi M. Toji T. (2023). Periostin secreted by cancer-associated fibroblasts promotes cancer progression and drug resistance in non-small cell lung cancer. J. Mol. Med. 101, 1603–1614. 10.1007/s00109-023-02384-7 37831111

[B107] Tay R. E. Richardson E. K. Toh H. C. (2021). Revisiting the role of CD4+ T cells in cancer immunotherapy-new insights into old paradigms. Cancer Gene Ther. 28, 5–17. 10.1038/s41417-020-0183-x 32457487 PMC7886651

[B108] Uhlen M. Zhang C. Lee S. Sjöstedt E. Fagerberg L. Bidkhori G. (2017). A pathology atlas of the human cancer transcriptome. Science 357, eaan2507. 10.1126/science.aan2507 28818916

[B109] Valdembri D. Serini G. (2021). The roles of integrins in cancer. Fac. Rev. 10, 45. 10.12703/r/10-45 34131655 PMC8170687

[B110] van Splunder H. Villacampa P. Martínez-Romero A. Graupera M. (2024). Pericytes in the disease spotlight. Trends Cell Biol. 34, 58–71. 10.1016/j.tcb.2023.06.001 37474376 PMC10777571

[B111] Wang D. Jiao C. Zhu Y. Liang D. Zao M. Meng X. (2017). Activation of CXCL12/CXCR4 renders colorectal cancer cells less sensitive to radiotherapy via up-regulating the expression of survivin. Exp. Biol. Med. 242, 429–435. 10.1177/1535370216675068 PMC5298539 27798120

[B112] Wang F. Long J. Li L. Wu Z.-X. Da T.-T. Wang X.-Q. (2023). Single-cell and spatial transcriptome analysis reveals the cellular heterogeneity of liver metastatic colorectal cancer. Sci. Adv. 9, eadf5464. 10.1126/sciadv.adf5464 37327339 PMC10275599

[B113] Wang H. Ren R. Yang Z. Cai J. Du S. Shen X. (2021b). The COL11A1/Akt/CREB signaling axis enables mitochondrial-mediated apoptotic evasion to promote chemoresistance in pancreatic cancer cells through modulating BAX/BCL-2 function. J. Cancer 12, 1406–1420. 10.7150/jca.47032 33531986 PMC7847647

[B114] Wang M. Wu C.-P. Pan J.-Y. Zheng W.-W. Cao X.-J. Fan G.-K. (2015). Cancer-associated fibroblasts in a human HEp-2 established laryngeal xenografted tumor are not derived from cancer cells through epithelial-mesenchymal transition, phenotypically activated but karyotypically normal. PLoS One 10, e0117405. 10.1371/journal.pone.0117405 25658113 PMC4319834

[B115] Wang S. Wang J. Chen Z. Luo J. Guo W. Sun L. (2024). Targeting M2-like tumor-associated macrophages is a potential therapeutic approach to overcome antitumor drug resistance. NPJ Precis. Oncol. 8, 31. 10.1038/s41698-024-00522-z 38341519 PMC10858952

[B116] Wang W. Kryczek I. Dostál L. Lin H. Tan L. Zhao L. (2016). Effector T cells abrogate stroma-mediated chemoresistance in ovarian cancer. Cell. 165, 1092–1105. 10.1016/j.cell.2016.04.009 27133165 PMC4874853

[B117] Wang Y. Liang Y. Xu H. Zhang X. Mao T. Cui J. (2021a). Single-cell analysis of pancreatic ductal adenocarcinoma identifies a novel fibroblast subtype associated with poor prognosis but better immunotherapy response. Cell Discov. 7, 36. 10.1038/s41421-021-00271-4 34035226 PMC8149399

[B118] Wang Z. An J. Zhu D. Chen H. Lin A. Kang J. (2022). Periostin: an emerging activator of multiple signaling pathways. J. Cell Commun. Signal 16, 515–530. 10.1007/s12079-022-00674-2 35412260 PMC9733775

[B119] Wei L. Ye H. Li G. Lu Y. Zhou Q. Zheng S. (2018). Cancer-associated fibroblasts promote progression and gemcitabine resistance via the SDF-1/SATB-1 pathway in pancreatic cancer. Cell Death Dis. 9, 1065. 10.1038/s41419-018-1104-x 30337520 PMC6194073

[B120] Werba G. Weissinger D. Kawaler E. A. Zhao E. Kalfakakou D. Dhara S. (2023). Single-cell RNA sequencing reveals the effects of chemotherapy on human pancreatic adenocarcinoma and its tumor microenvironment. Nat. Commun. 14, 797. 10.1038/s41467-023-36296-4 36781852 PMC9925748

[B121] Wilke C. M. Kryczek I. Zou W. (2011). Antigen-presenting cell (APC) subsets in ovarian cancer. Int. Rev. Immunol. 30, 120–126. 10.3109/08830185.2011.567362 21557638

[B122] Wu J. Liu X. Wu J. Lou C. Zhang Q. Chen H. (2022). CXCL12 derived from CD248-expressing cancer-associated fibroblasts mediates M2-polarized macrophages to promote nonsmall cell lung cancer progression. Biochimica Biophysica Acta (BBA) - Mol. Basis Dis. 1868, 166521. 10.1016/j.bbadis.2022.166521 35985448

[B123] Wu S. Z. Roden D. L. Wang C. Holliday H. Harvey K. Cazet A. S. (2020). Stromal cell diversity associated with immune evasion in human triple‐negative breast cancer. EMBO J. 39, e104063. 10.15252/embj.2019104063 32790115 PMC7527929

[B124] Wu X. Ren Y. Yao R. Zhou L. Fan R. (2021). Circular RNA circ-MMP11 contributes to lapatinib resistance of breast cancer cells by regulating the miR-153-3p/ANLN Axis. Front. Oncol. 11, 639961. 10.3389/fonc.2021.639961 34295807 PMC8290203

[B125] Wu Y.-H. Huang Y.-F. Chang T.-H. Chou C.-Y. (2017). Activation of TWIST1 by COL11A1 promotes chemoresistance and inhibits apoptosis in ovarian cancer cells by modulating NF-κB-mediated IKKβ expression. Int. J. Cancer 141, 2305–2317. 10.1002/ijc.30932 28815582

[B126] Wu Y.-H. Huang Y.-F. Chen C.-C. Chou C.-Y. (2019). Akt inhibitor SC66 promotes cell sensitivity to cisplatin in chemoresistant ovarian cancer cells through inhibition of COL11A1 expression. Cell Death Dis. 10, 322. 10.1038/s41419-019-1555-8 30975980 PMC6459878

[B127] Xiao Z.-M. Wang X.-Y. Wang A.-M. (2015). Periostin induces chemoresistance in colon cancer cells through activation of the PI3K/Akt/survivin pathway. Biotechnol. Appl. Biochem. 62, 401–406. 10.1002/bab.1193 24372557

[B128] Xing H. Cao Y. Weng D. Tao W. Song X. Wang W. (2008). Fibronectin-mediated activation of Akt2 protects human ovarian and breast cancer cells from docetaxel-induced apoptosis via inhibition of the p38 pathway. Apoptosis 13, 213–223. 10.1007/s10495-007-0158-5 18158623

[B129] Xu A. Xu X.-N. Luo Z. Huang X. Gong R.-Q. Fu D.-Y. (2023). Identification of prognostic cancer-associated fibroblast markers in luminal breast cancer using weighted gene co-expression network analysis. Front. Oncol. 13, 1191660. 10.3389/fonc.2023.1191660 37207166 PMC10191114

[B130] Yang W. Zhang S. Li T. Zhou Z. Pan J. (2022). Single-cell analysis reveals that cancer-associated fibroblasts stimulate oral squamous cell carcinoma invasion via the TGF-β/Smad pathway. Acta Biochim. Biophys. Sin. 55, 262–273. 10.3724/abbs.2022132 36148955 PMC10157546

[B131] Ye Y. Zhang R. Feng H. (2020). Fibronectin promotes tumor cells growth and drugs resistance through a CDC42-YAP-dependent signaling pathway in colorectal cancer. Cell Biol. Int. 44, 1840–1849. 10.1002/cbin.11390 32437085

[B132] Yeung T.-L. Leung C. S. Wong K.-K. Samimi G. Thompson M. S. Liu J. (2013). TGF-β modulates ovarian cancer invasion by upregulating CAF-derived versican in the tumor microenvironment. Cancer Res. 73, 5016–5028. 10.1158/0008-5472.CAN-13-0023 23824740 PMC3745588

[B133] Yoon H. Tang C.-M. Banerjee S. Delgado A. L. Yebra M. Davis J. (2021). TGF-β1-mediated transition of resident fibroblasts to cancer-associated fibroblasts promotes cancer metastasis in gastrointestinal stromal tumor. Oncogenesis 10, 13. 10.1038/s41389-021-00302-5 33568624 PMC7876107

[B134] You T. Tang H. Wu W. Gao J. Li X. Li N. (2023). POSTN secretion by extracellular matrix cancer-associated fibroblasts (eCAFs) correlates with poor ICB response via macrophage chemotaxis activation of Akt signaling pathway in gastric cancer. Aging Dis. 14, 2177–2192. 10.14336/AD.2023.0503 37199594 PMC10676785

[B135] Yu G. Chen W. Li X. Yu L. Xu Y. Ruan Q. (2022). TWIST1-EP300 expedites gastric cancer cell resistance to apatinib by activating the expression of COL1A2. Anal. Cell Pathol. 2022, 5374262. 10.1155/2022/5374262 PMC8888114 35242497

[B136] Yu X. Shi W. Zhang Y. Wang X. Sun S. Song Z. (2017). CXCL12/CXCR4 axis induced miR-125b promotes invasion and confers 5-fluorouracil resistance through enhancing autophagy in colorectal cancer. Sci. Rep. 7, 42226. 10.1038/srep42226 28176874 PMC5296742

[B137] Zha H. Wang X. Zhu Y. Chen D. Han X. Yang F. (2019). Intracellular activation of complement C3 leads to PD-L1 antibody treatment resistance by modulating tumor-associated macrophages. Cancer Immunol. Res. 7, 193–207. 10.1158/2326-6066.CIR-18-0272 30514794

[B138] Zhang C. Fei Y. Wang H. Hu S. Liu C. Hu R. (2023a). CAFs orchestrates tumor immune microenvironment-A new target in cancer therapy? Front. Pharmacol. 14, 1113378. 10.3389/fphar.2023.1113378 37007004 PMC10064291

[B139] Zhang F. Cui J.-Y. Gao H.-F. Yu H. Gao F.-F. Chen J.-L. (2020b). Cancer-associated fibroblasts induce epithelial-mesenchymal transition and cisplatin resistance in ovarian cancer via CXCL12/CXCR4 axis. Future Oncol. 16, 2619–2633. 10.2217/fon-2020-0095 32804554

[B140] Zhang H. Wu H. Guan J. Wang L. Ren X. Shi X. (2015). Paracrine SDF-1α signaling mediates the effects of PSCs on GEM chemoresistance through an IL-6 autocrine loop in pancreatic cancer cells. Oncotarget 6, 3085–3097. 10.18632/oncotarget.3099 25609203 PMC4413639

[B141] Zhang H. Zhao Y. Liu X. Fu L. Gu F. Ma Y. (2021c). High expression of complement component C7 indicates poor prognosis of breast cancer and is insensitive to taxane-anthracycline chemotherapy. Front. Oncol. 11, 724250. 10.3389/fonc.2021.724250 34631552 PMC8497743

[B142] Zhang J. Zhang J. Wang F. Xu X. Li X. Guan W. (2021d). Overexpressed COL5A1 is correlated with tumor progression, paclitaxel resistance, and tumor-infiltrating immune cells in ovarian cancer. J. Cell Physiol. 236, 6907–6919. 10.1002/jcp.30350 33655494

[B143] Zhang M. Yang H. Wan L. Wang Z. Wang H. Ge C. (2020a). Single-cell transcriptomic architecture and intercellular crosstalk of human intrahepatic cholangiocarcinoma. J. Hepatol. 73, 1118–1130. 10.1016/j.jhep.2020.05.039 32505533

[B144] Zhang S. Fang W. Zhou S. Zhu D. Chen R. Gao X. (2023b). Single cell transcriptomic analyses implicate an immunosuppressive tumor microenvironment in pancreatic cancer liver metastasis. Nat. Commun. 14, 5123. 10.1038/s41467-023-40727-7 37612267 PMC10447466

[B145] Zhang X. Peng L. Luo Y. Zhang S. Pu Y. Chen Y. (2021a). Dissecting esophageal squamous-cell carcinoma ecosystem by single-cell transcriptomic analysis. Nat. Commun. 12, 5291. 10.1038/s41467-021-25539-x 34489433 PMC8421382

[B146] Zhang Y. Wang D. Peng M. Tang L. Ouyang J. Xiong F. (2021b). Single-cell RNA sequencing in cancer research. J. Exp. Clin. Cancer Res. 40, 81. 10.1186/s13046-021-01874-1 33648534 PMC7919320

[B147] Zhao Y. Mei S. Huang Y. Chen J. Zhang X. Zhang P. (2022). Integrative analysis deciphers the heterogeneity of cancer-associated fibroblast and implications on clinical outcomes in ovarian cancers. Comput. Struct. Biotechnol. J. 20, 6403–6411. 10.1016/j.csbj.2022.11.025 36420154 PMC9679440

[B148] Zhu M. Ye C. Wang J. Yang G. Ying X. (2021). Activation of COL11A1 by PRRX1 promotes tumor progression and radioresistance in ovarian cancer. Int. J. Radiat. Biol. 97, 958–967. 10.1080/09553002.2021.1928780 33970764

