# Supplementary material for: Exploring the diversity of cancer-associated fibroblasts: insights into mechanisms of drug resistance
Source: Front Cell Dev Biol. 2024 May 16;12:1403122. doi: 10.3389/fcell.2024.1403122 (PMC11137237; doi:10.3389/fcell.2024.1403122)
Supplement: Supplementary file 1 [file DataSheet1.PDF]

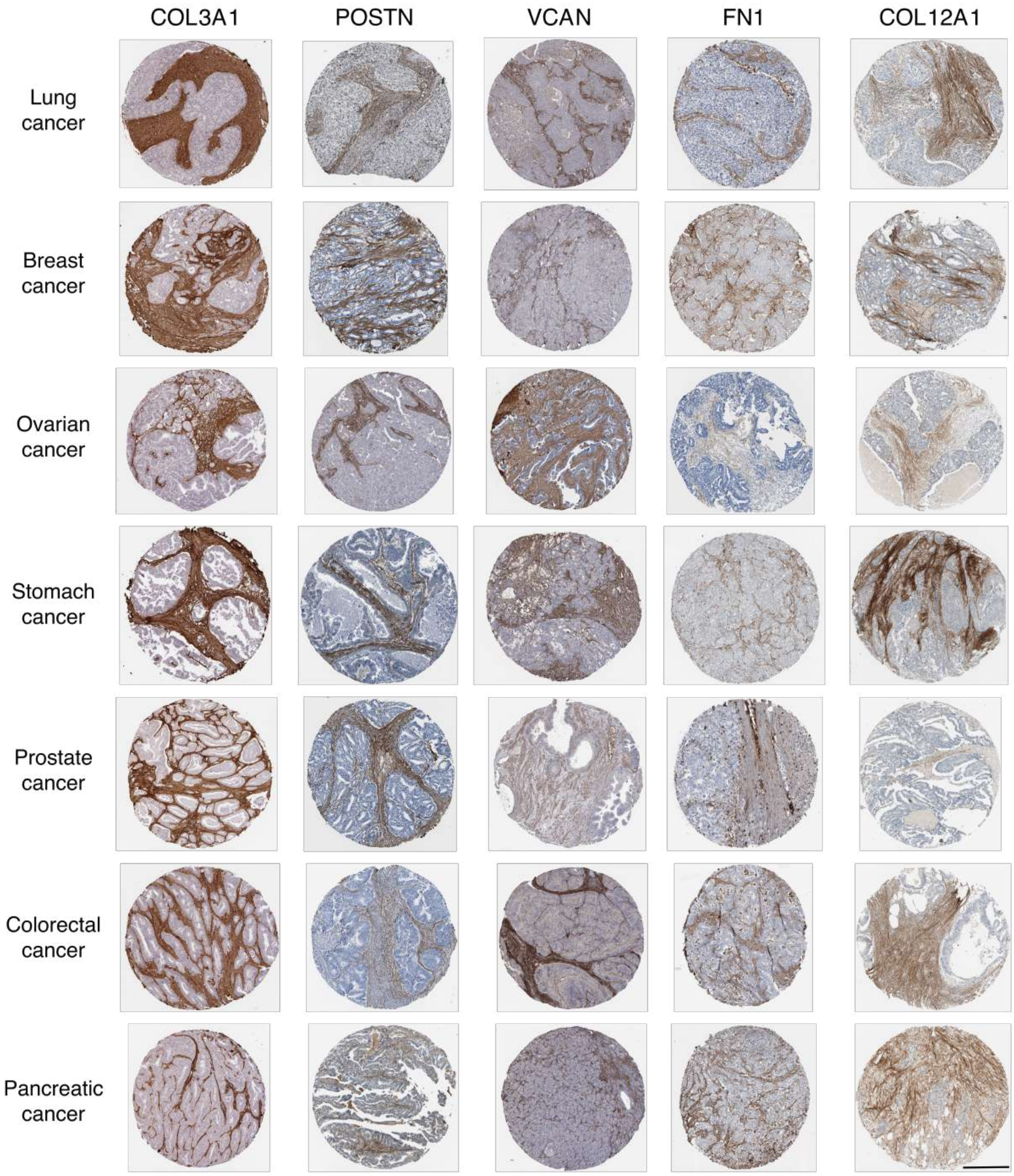

Supplementary Figure S1. Immunohistochemistry (IHC) staining images showing protein expressions of COL3A1, POSTN, VCAN, FN1, and COL12A1 in different tumor types from the Human Protein Atlas database. Scale bar = 400 μm.

Supplementary Table S1. Expression changes of myCAFs markers in the tumor compared to normal tissue. Up mRNA - upregulation at the gene level, Down mRNA - downregulation at the gene level, Up protein - upregulation at the protein level, Down protein - downregulation at the protein level. Red indicates increased expression of the gene/protein in the tumor compared to normal tissue, blue indicates decreased expression, and purple indicates both increased and decreased expression observed in different studies.

Cancer	Lung cancer	Breast cancer	Ovarian cancer	Stomach cancer	Prostate cancer	Colorectal cancer	Pancreatic cancer	Bladder cancer
COL11A1	Up mRNA [1–3]	Up mRNA [1] Up protein [4]	Up mRNA [1]	Up mRNA [1,5]	-	Up mRNA [1]	Up mRNA [1,6] Up protein [6]	Up mRNA
COL10A1	Up mRNA [1,3]	Up mRNA [1] Up protein [7]	Up mRNA [1]	Up mRNA [1,5]	Up mRNA [1]	Up mRNA [1]	Up mRNA [1]	Up mRNA [1] Up protein [8]
CTHRC1	Up mRNA [1]	Up mRNA [1]	Up mRNA [1]	Up mRNA [1,5]	Up mRNA [9] Up protein [9]	Up mRNA [1,10] Up protein [10,11]	Up mRNA [1]	Up mRNA [12]
COL5A1	-	Up mRNA [1]	Up mRNA [13] Down mRNA [1]	Up mRNA [1,14]	Down mRNA [1]	-	Up mRNA [1]	-
COL1A1	Up mRNA [1,3,15] Up protein [15]	Up mRNA [1]	Up protein [16,17]	Up mRNA [1,14]	-	Up protein [18,19]	Up mRNA [1]	-
COL3A1	Up mRNA [1,3,20]	Up mRNA [1]	-	Up mRNA [1,14]	-	Up mRNA [19]	Up mRNA [1]	Up mRNA [21] Up protein [21]
COL12A1	-	Up mRNA [22]	Down mRNA [1]	Up mRNA [1,23] Up protein [23]	-	Up protein [24]	Up mRNA [1]	-
COL8A1	Up mRNA [1]	Up mRNA [1,25]	-	Up mRNA [1,5,26] Up protein [26]	-	-	Up mRNA [1]	-
COL1A2	-	Up mRNA [1]	-	Up mRNA [1,5,14]	-	Up mRNA [19] Up protein [27]	Up mRNA [1]	-
COL5A2	-	Up mRNA [1]	Down mRNA [1]	Up mRNA [1] Up protein [28]	Down mRNA [1]	Up mRNA [29] Up protein [29] Down mRNA [1]	Up mRNA [1]	-

MMP11	Up mRNA [1,3]	Up mRNA [1,30]	Up mRNA [1]	Up mRNA [1]	Up mRNA [31,32] Up protein [31,32]	Up mRNA [1,33]	Up mRNA [1]	Up mRNA [1,34]
POSTN	Up mRNA [1]	Up mRNA [1]	Up mRNA [1,35] Up protein [35]	Up mRNA [36]	Up mRNA [37]	Up protein [38] Up mRNA [38]	Up mRNA [1] Up protein [39]	-
VCAN	-	Up mRNA [1]	Up mRNA [1]	Up mRNA [1]	-	Up mRNA [40]	Up mRNA [1]	-
FN1	Down mRNA [1]	Up mRNA [1]	-	Up mRNA [1,14,41] Up protein [41]	Down mRNA [1]	Down mRNA [1]	Up mRNA [1]	-
INHBA	-	Up mRNA [1]	Up mRNA [42] Up protein [42]	Up mRNA [1]	-	Up mRNA [1]	Up mRNA [1]	Up mRNA [1]
THBS2	-	-	Down mRNA [1] Up protein [16]	Up mRNA [1,5]	-	Up mRNA [1,43]	Up mRNA [1]	-
SULF1	Up mRNA [1,3]	Up mRNA [1]	Up mRNA [1]	Up mRNA [1,44–46]	-	-	Up mRNA [1] Up protein [47]	-

Supplementary Table S2. Prognostic impact of myCAF markers in various types of tumors. Bad - increased gene expression associated with poor prognosis, Good - increased gene expression associated with good prognosis. Blue indicates an association of increased gene expression with poor prognosis, red indicates an association with good prognosis.

Cancer	Lung cancer	Breast cancer	Ovarian cancer	Stomach cancer	Prostate cancer	Colorectal cancer	Pancreatic cancer	Bladder cancer
COL11A1	Bad [1]	-	-	Bad [1]	-	-	-	Bad [1]
COL10A1	-	-	-	-	-	-	-	Bad [1]
CTHRC1	-	Bad [1]	-	Bad [1]	-	Bad [1]	-	Bad [1]
COL5A1	-	-	Bad [13]	-	-	-	-	Bad [1]
COL1A1	-	Bad [48]	-	Bad [1]	-	-	-	Bad [1]
COL3A1	-	-	-	Bad [1]	-	-	-	Bad [1]
COL12A1	Bad [1]	-	-	Bad [1]	-	-	-	-
COL8A1	-	-	-	Bad [1]	-	-	-	-
COL1A2	Bad [1]	-	-	Bad [1]	-	-	-	Bad [1]
COL5A2	Bad [1]	-	-	Bad [28]	-	-	Bad [1]	Bad [1]
MMP11	-	-	-	-	-	-	-	Bad [1]
POSTN	Bad [1]	Bad [49]	-	-	-	Bad [1]	-	Bad [1]
VCAN	Bad [1]	-	Bad [50]	Bad [1,51]	-	-	-	Bad [1]
FN1	-	Bad [52]	-	Bad [1]	-	-	-	Bad [1]
INHBA	-	Bad [1,53]	-	-	-	-	Bad [1]	-
THBS2	Bad [1]	-	-	-	-	Bad [43]	-	-
SULF1	-	-	-	-	-	-	-	Bad [1]

Supplementary Table S3. Expression changes of iCAFs markers in the tumor compared to normal tissue. Up mRNA - upregulation at the gene level, Down mRNA - downregulation at the gene level, Up protein - upregulation at the protein level, Down protein - downregulation at the protein level. Red indicates increased expression of the gene/protein in the tumor compared to normal tissue, blue indicates decreased expression, and purple indicates both increased and decreased expression observed in different studies.

Cancer	Lung cancer	Breast cancer	Ovarian cancer	Stomach cancer	Prostate cancer	Colorectal cancer	Pancreatic cancer	Bladder cancer
CFD	Down mRNA [1,54]	Down mRNA [1,55]	Down mRNA [1]	Down mRNA [1]	Down mRNA [1]	Down mRNA [1,56] Up mRNA [57]	-	Down mRNA [1]
C3	Down mRNA [1]	Down mRNA [1]	Up mRNA [1]	Up mRNA [1,58] Up protein [58]	-	Down mRNA [1] Up mRNA [59]	Up mRNA [1]	Down mRNA [1]
C7	Down mRNA [1,60]	Down mRNA [1] Up mRNA [61]	Down mRNA [1,60]	Down mRNA [1]	Down mRNA [1,62]	Down mRNA [1] Up mRNA [57]	-	Down mRNA [1]
CXCL12	Down mRNA [1]	Down mRNA [1] Down protein [63]	Down mRNA [1]	-	-	Down mRNA [1]	-	Down mRNA [1]
CXCL14	Up mRNA [1]	Down mRNA [1]	Up mRNA [1,64] Up protein [64]	Up mRNA [1]	Up mRNA [1]	Up mRNA [1] Down mRNA [65] Down protein [65]	Up mRNA [1]	Up mRNA [1]
FBLN1	Down mRNA [1,66] Up protein [67]	Down mRNA [1]	Down mRNA [1]	-	Down mRNA [1]	Down mRNA [1]	Up mRNA [1]	Down mRNA [1,68] Down protein [68]
DPT	Down mRNA [1]	Down mRNA [1,69]	Down mRNA [1] Up mRNA [70]	Down mRNA [1]	Down mRNA [1]	Down mRNA [1]	-	Down mRNA [1]
APOD	Down mRNA [1]	Down mRNA [1,71]	Down mRNA [1]	Down mRNA [1] Up mRNA [72]	Up protein [73]	Down mRNA [1]	Up mRNA [1]	Down mRNA [1]
IGF1	Down mRNA [1]	Down mRNA [1]	-	-	Down mRNA [1]	Down mRNA [1]	-	Down mRNA [1]
IGFBP6	Down	Down	Down	Down	Down	Down	Up mRNA	Down

	mRNA [1]	mRNA [1]	mRNA [1]	mRNA [1]	mRNA [1]	mRNA [1]	[1]	mRNA [1]
PTGDS	Down mRNA [1]	Down mRNA [1]	Up mRNA [1]	-	Down mRNA [1]	Down mRNA [1]	Up mRNA [1]	Down mRNA [1]
GSN	-	Down mRNA [1]	-	-	-	Down mRNA [1]	Up mRNA [1]	Down mRNA [1]

Supplementary Table S4. Prognostic impact of iCAF markers in various types of tumors. Bad - increased gene expression associated with poor prognosis, Good - increased gene expression associated with good prognosis. Blue indicates an association of increased gene expression with poor prognosis, red indicates an association with good prognosis.

Cancer	Lung cancer	Breast cancer	Ovarian cancer	Stomach cancer	Prostate cancer	Colorectal cancer	Pancreatic cancer	Bladder cancer
CFD	-	-	-	-	-	-	-	-
C3	-	-	-	-	-	-	-	-
C7	Good [1]	Bad [61]	-	Bad [1]	-	-	-	Bad [1]
CXCL12	-	-	Bad [74]	-	-	-	Bad [75]	-
CXCL14	-	Good [1]	-	-	-	-	-	-
FBLN1	-	-	-	Bad [1]	Good [1]	-	-	-
DPT	-	-	-	-	-	-	-	-
APOD	-	Good [1]	-	Bad [1]	-	-	-	Bad [1]
IGF1	-	-	-	Bad [1]	Good [1]	-	-	-
IGFBP6	-	-	-	Bad [1]	-	-	-	-
PTGDS	Good [1]	-	-	-	-	-	-	-
GSN	-	-	-	-	-	-	-	Bad [1]

References

1. Tang Z, Kang B, Li C, Chen T, Zhang Z. GEPIA2: an enhanced web server for large-scale expression profiling and interactive analysis. Nucleic Acids Res. 2019;47: W556–W560.

2. Shen L, Yang M, Lin Q, Zhang Z, Zhu B, Miao C. COL11A1 is overexpressed in recurrent non-small cell lung cancer and promotes cell proliferation, migration, invasion and drug resistance. Oncol Rep. 2016;36: 877–885.

3. Yang H, Jiang P, Liu D, Wang H-Q, Deng Q, Niu X, et al. Matrix Metalloproteinase 11 Is a Potential Therapeutic Target in Lung Adenocarcinoma. Mol Ther Oncolytics. 2019;14: 82–93.

4. Luo Q, Li J, Su X, Tan Q, Zhou F, Xie S. COL11A1 serves as a biomarker for poor prognosis and correlates with immune infiltration in breast cancer. Front Genet. 2022;13: 935860.

5. Sun C, Chen Y, Kim NH, Lowe S, Ma S, Zhou Z, et al. Identification and Verification of Potential Biomarkers in Gastric Cancer By Integrated Bioinformatic Analysis. Front Genet. 2022;13: 911740.

6. Ma G, Li G, Fan W, Xu Y, Song S, Guo K, et al. Circ-0005105 activates COL11A1 by targeting miR-20a-3p to promote pancreatic ductal adenocarcinoma progression. Cell Death Dis. 2021;12: 656.

7. Zhou W, Li Y, Gu D, Xu J, Wang R, Wang H, et al. High expression COL10A1 promotes breast cancer progression and predicts poor prognosis. Heliyon. 2022;8: e11083.

8. Wang X, Bai Y, Zhang F, Li D, Chen K, Wu R, et al. Prognostic value of COL10A1 and its correlation with tumor-infiltrating immune cells in urothelial bladder cancer: A comprehensive study based on bioinformatics and clinical analysis validation. Front Immunol. 2023;14: 955949.

9. Ma Z, Chao F, Wang S, Song Z, Zhuo Z, Zhang J, et al. CTHRC1 affects malignant tumor cell behavior and is regulated by miR-30e-5p in human prostate cancer. Biochem Biophys Res Commun. 2020;525: 418–424.

10. Liu Y, Chen X, Xu Y, Yang T, Wang H, Wang Z, et al. CTHRC1 promotes colorectal cancer progression by recruiting tumor-associated macrophages via up-regulation of CCL15. J Mol Med . 2023. doi:10.1007/s00109-023-02399-0

11. Pang C, Wang H, Shen C, Liang H. Application Potential of as a Diagnostic and Prognostic Indicator for Colon Adenocarcinoma. Front Mol Biosci. 2022;9: 849771.

12. Li Y, Cheng X, Yan J, Jiang S. CTHRC1 facilitates bladder cancer cell proliferation and invasion through regulating the PI3K/Akt signaling pathway. Arch Med Sci. 2022;18: 183–194.

13. Zhang J, Zhang J, Wang F, Xu X, Li X, Guan W, et al. Overexpressed COL5A1 is correlated with tumor progression, paclitaxel resistance, and tumor-infiltrating immune cells in ovarian cancer. J Cell Physiol. 2021;236: 6907–6919.

14. Ucaryilmaz Metin C, Ozcan G. Comprehensive bioinformatic analysis reveals a cancer-associated fibroblast gene signature as a poor prognostic factor and potential therapeutic target in gastric cancer. BMC Cancer. 2022;22: 692.

15. Hou L, Lin T, Wang Y, Liu B, Wang M. Collagen type 1 alpha 1 chain is a novel

predictive biomarker of poor progression-free survival and chemoresistance in metastatic lung cancer. J Cancer. 2021;12: 5723–5731.

16. Wang M, Wang J, Liu J, Zhu L, Ma H, Zou J, et al. Systematic prediction of key genes for ovarian cancer by co-expression network analysis. J Cell Mol Med. 2020;24: 6298–6307.

17. Li M, Wang J, Wang C, Xia L, Xu J, Xie X, et al. Microenvironment remodeled by tumor and stromal cells elevates fibroblast-derived COL1A1 and facilitates ovarian cancer metastasis. Exp Cell Res. 2020;394: 112153.

18. Zhang Z, Wang Y, Zhang J, Zhong J, Yang R. COL1A1 promotes metastasis in colorectal cancer by regulating the WNT/PCP pathway. Mol Med Rep. 2018;17: 5037–5042.

19. Hosseini ST, Nemati F. Identification of GUCA2A and COL3A1 as prognostic biomarkers in colorectal cancer by integrating analysis of RNA-Seq data and qRT-PCR validation. Sci Rep. 2023;13: 17086.

20. Wang L, Sun Y, Guo Z, Liu H. COL3A1 Overexpression Associates with Poor Prognosis and Cisplatin Resistance in Lung Cancer. Balkan Med J. 2022;39: 393–400.

21. Yuan L, Shu B, Chen L, Qian K, Wang Y, Qian G, et al. Overexpression of COL3A1 confers a poor prognosis in human bladder cancer identified by co-expression analysis. Oncotarget. 2017;8: 70508–70520.

22. COL12A1 as a prognostic biomarker in HER2-enriched breast cancer and its association with immune infiltration. Eur J Gynaecol Oncol. 2022. doi:10.22514/ejgo.2022.045

23. Jiang X, Wu M, Xu X, Zhang L, Huang Y, Xu Z, et al. COL12A1, a novel potential prognostic factor and therapeutic target in gastric cancer. Mol Med Rep. 2019;20: 3103–3112.

24. Wu Y, Xu Y. Integrated bioinformatics analysis of expression and gene regulation network of COL12A1 in colorectal cancer. Cancer Med. 2020;9: 4743–4755.

25. Peng W, Li J-D, Zeng J-J, Zou X-P, Tang D, Tang W, et al. Clinical value and potential mechanisms of COL8A1 upregulation in breast cancer: a comprehensive analysis. Cancer Cell Int. 2020;20: 392.

26. She Y, Zhao X, Wu P, Xue L, Wan S, Zhang L, et al. COL8A1 Predicts the Clinical Prognosis of Gastric Cancer and Is Related to Epithelial-Mesenchymal Transition. Biomed Res Int. 2022;2022: 7567447.

27. Yuan X, He Y, Wang W. ceRNA network-regulated COL1A2 high expression correlates with poor prognosis and immune infiltration in colon adenocarcinoma. Sci Rep. 2023;13: 16932.

28. Chen M, Zhu X, Zhang L, Zhao D. COL5A2 is a prognostic-related biomarker and correlated with immune infiltrates in gastric cancer based on transcriptomics and single-cell RNA sequencing. BMC Med Genomics. 2023;16: 220.

29. Wang J, Jiang Y-H, Yang P-Y, Liu F. Increased Collagen Type V α2 (COL5A2) in Colorectal Cancer is Associated with Poor Prognosis and Tumor Progression. Onco Targets Ther. 2021;14: 2991–3002.

30. Belachew EB, Desta AF, Deneke DB, Gebremariam TY, Tefera DA, Atire FA, et al. The expression of matrix metalloproteinase 2, 9 and 11 in Ethiopian breast cancer patients. BMC Res Notes. 2023;16: 253.

31. Tan B, Zheng X, Xie X, Chen Y, Li Y, He W. MMP11 and MMP14 contribute to the interaction between castration-resistant prostate cancer and adipocytes. Am J Cancer Res. 2023;13: 5934–5949.

32. Eiro N, Fernández-Gómez JM, Gonzalez-Ruiz de León C, Fraile M, Gonzalez-Suarez J, Lobo-Rodríguez B, et al. Gene Expression Profile of Stromal Factors in Cancer-Associated Fibroblasts from Prostate Cancer. Diagnostics (Basel). 2022;12. doi:10.3390/diagnostics12071605

33. Huang H-C, Shiu B-H, Su S-C, Huang C-C, Ting W-C, Chang L-C, et al. The Impact of Matrix Metalloproteinase-11 Polymorphisms on Colorectal Cancer Progression and Clinicopathological Characteristics. Diagnostics (Basel). 2022;12. doi:10.3390/diagnostics12071685

34. Shen C, Da L, Wu Z, Wang Y, Gao S, Tian D, et al. Expression and Prognostic Significance of the MMP Family Molecules in Bladder Cancer. Comb Chem High Throughput Screen. 2021;24: 1183–1196.

35. Chu L, Wang F, Zhang W, Li H-F, Xu J, Tong X-W. Periostin Secreted by Carcinoma-Associated Fibroblasts Promotes Ovarian Cancer Cell Platinum Resistance Through the PI3K/Akt Signaling Pathway. Technol Cancer Res Treat. 2020;19: 1533033820977535.

36. Lu S, Peng L, Ma F, Chai J, Hua Y, Yang W, et al. Increased Expression of POSTN Predicts Poor Prognosis: a Potential Therapeutic Target for Gastric Cancer. J Gastrointest Surg. 2023;27: 233–249.

37. Nuzzo PV, Rubagotti A, Zinoli L, Ricci F, Salvi S, Boccardo S, et al. The prognostic value of periostin expression in human prostate cancer. Ann Oncol. 2012;23: ix301.

38. Liu T, Xia R, Li C, Chen X, Cai X, Li W. mRNA expression level of , , , and modulates 5-fluorouracil resistance in colon cancer cells. Exp Ther Med. 2021;22: 1023.

39. Liu Y, Li F, Gao F, Xing L, Qin P, Liang X, et al. Periostin promotes the chemotherapy resistance to gemcitabine in pancreatic cancer. Tumour Biol. 2016;37: 15283–15291.

40. Chida S, Okayama H, Noda M, Saito K, Nakajima T, Aoto K, et al. Stromal VCAN expression as a potential prognostic biomarker for disease recurrence in stage II-III colon cancer. Carcinogenesis. 2016;37: 878–887.

41. Sun Y, Zhao C, Ye Y, Wang Z, He Y, Li Y, et al. High expression of fibronectin 1 indicates poor prognosis in gastric cancer. Oncol Lett. 2020;19: 93–102.

42. Li X, Yang Z, Xu S, Wang Z, Jin P, Yang X, et al. Targeting INHBA in Ovarian Cancer Cells Suppresses Cancer Xenograft Growth by Attenuating Stromal Fibroblast Activation. Dis Markers. 2019;2019: 7275289.

43. Wang X, Zhang L, Li H, Sun W, Zhang H, Lai M. THBS2 is a Potential Prognostic Biomarker in Colorectal Cancer. Sci Rep. 2016;6: 33366.

44. Hu Y, Li J, Luo H, Song W, Yang J. Differential Expression of COL1A1, COL1A2,

COL6A3, and SULF1 as Prognostic Biomarkers in Gastric Cancer. Int J Gen Med. 2021;14: 5835–5843.

45. Hur K, Han T-S, Jung E-J, Yu J, Lee H-J, Kim WH, et al. Up-regulated expression of sulfatases (SULF1 and SULF2) as prognostic and metastasis predictive markers in human gastric cancer. J Pathol. 2012;228: 88–98.

46. Junnila S, Kokkola A, Mizuguchi T, Hirata K, Karjalainen-Lindsberg M-L, Puolakkainen P, et al. Gene expression analysis identifies over-expression of CXCL1, SPARC, SPP1, and SULF1 in gastric cancer. Genes Chromosomes Cancer. 2010;49: 28–39.

47. Lyu Y, Cheng Y, Wang B, Chen L, Zhao S. Sulfatase 1 expression in pancreatic cancer and its correlation with clinicopathological features and postoperative prognosis. Cancer Biomark. 2018;22: 701–707.

48. Liu J, Shen J-X, Wu H-T, Li X-L, Wen X-F, Du C-W, et al. Collagen 1A1 (COL1A1) promotes metastasis of breast cancer and is a potential therapeutic target. Discov Med. 2018;25: 211–223.

49. Oo KK, Kamolhan T, Soni A, Thongchot S, Mitrpant C, O-Charoenrat P, et al. Development of an engineered peptide antagonist against periostin to overcome doxorubicin resistance in breast cancer. BMC Cancer. 2021;21: 65.

50. Ghosh S, Albitar L, LeBaron R, Welch WR, Samimi G, Birrer MJ, et al. Up-regulation of stromal versican expression in advanced stage serous ovarian cancer. Gynecol Oncol. 2010;119: 114–120.

51. Song J, Wei R, Huo S, Liu C, Liu X. Versican enrichment predicts poor prognosis and response to adjuvant therapy and immunotherapy in gastric cancer. Front Immunol. 2022;13: 960570.

52. Saatci O, Kaymak A, Raza U, Ersan PG, Akbulut O, Banister CE, et al. Targeting lysyl oxidase (LOX) overcomes chemotherapy resistance in triple negative breast cancer. Nat Commun. 2020;11: 2416.

53. Liu M, Smith R, Liby T, Chiotti K, López CS, Korkola JE. INHBA is a mediator of aggressive tumor behavior in HER2+ basal breast cancer. Breast Cancer Res. 2022;24: 18.

54. Liu Z, Sun D, Zhu Q, Liu X. The screening of immune-related biomarkers for prognosis of lung adenocarcinoma. Bioengineered. 2021;12: 1273–1285.

55. Dong X, Yang Y, Xu G, Tian Z, Yang Q, Gong Y, et al. The initial expression alterations occurring to transcription factors during the formation of breast cancer: Evidence from bioinformatics. Cancer Med. 2022;11: 1371–1395.

56. Li Y-R, Meng K, Yang G, Liu B-H, Li C-Q, Zhang J-Y, et al. Diagnostic genes and immune infiltration analysis of colorectal cancer determined by LASSO and SVM machine learning methods: a bioinformatics analysis. J Gastrointest Oncol. 2022;13: 1188–1203.

57. Gardner IH, Siddharthan R, Watson K, Dewey E, Ruhl R, Khou S, et al. A Distinct Innate Immune Signature of Early Onset Colorectal Cancer. Immunohorizons. 2021;5: 489–499.

58. Yuan K, Ye J, Liu Z, Ren Y, He W, Xu J, et al. Complement C3 overexpression activates

JAK2/STAT3 pathway and correlates with gastric cancer progression. J Exp Clin Cancer Res. 2020;39: 9.

59. Liu Y, Wang X. Tumor microenvironment-associated gene C3 can predict the prognosis of colorectal adenocarcinoma: a study based on TCGA. Clin Transl Oncol. 2021;23: 1923–1933.

60. Ying L, Zhang F, Pan X, Chen K, Zhang N, Jin J, et al. Complement component 7 (C7), a potential tumor suppressor, is correlated with tumor progression and prognosis. Oncotarget. 2016;7: 86536–86546.

61. Zhang H, Zhao Y, Liu X, Fu L, Gu F, Ma Y. High Expression of Complement Component C7 Indicates Poor Prognosis of Breast Cancer and Is Insensitive to Taxane-Anthracycline Chemotherapy. Front Oncol. 2021;11: 724250.

62. Chen Z, Yan X, Du G-W, Tuoheti K, Bai X-J, Wu H-H, et al. Complement C7 (C7), a Potential Tumor Suppressor, Is an Immune-Related Prognostic Biomarker in Prostate Cancer (PC). Front Oncol. 2020;10: 1532.

63. Motyka J, Gacuta E, Kicman A, Kulesza M, Malinowski P, Ławicki S. CXCL12 and CXCR4 as Potential Early Biomarkers for Luminal A and Luminal B Subtypes of Breast Cancer. Cancer Manag Res. 2023;15: 573–589.

64. Gao L-N, Hao M, Liu X-H, Zhang L, Dong Y, Zhang Y-F, et al. CXCL14 facilitates the growth and metastasis of ovarian carcinoma cells via activation of the Wnt/β-catenin signaling pathway. J Ovarian Res. 2021;14: 159.

65. Lin K, Zou R, Lin F, Zheng S, Shen X, Xue X. Expression and effect of CXCL14 in colorectal carcinoma. Mol Med Rep. 2014;10: 1561–1568.

66. Fan H, Zhang J, Zou B, He Z. The Role of CEP55 Expression in Tumor Immune Response and Prognosis of Patients with Non-small Cell lung Cancer. Arch Iran Med. 2022;25: 432–442.

67. Wei S, Liu W, Xu M, Qin H, Liu C, Zhang R, et al. Cathepsin F and Fibulin-1 as novel diagnostic biomarkers for brain metastasis of non-small cell lung cancer. Br J Cancer. 2022;126: 1795–1805.

68. Xiao W, Wang J, Li H, Xia D, Yu G, Yao W, et al. Fibulin-1 is epigenetically down-regulated and related with bladder cancer recurrence. BMC Cancer. 2014;14: 677.

69. Ye D, Wang Y, Deng X, Zhou X, Liu D, Zhou B, et al. DNMT3a-dermatopontin axis suppresses breast cancer malignancy via inactivating YAP. Cell Death Dis. 2023;14: 106.

70. Pan S, Cheng L, White JT, Lu W, Utleg AG, Yan X, et al. Quantitative proteomics analysis integrated with microarray data reveals that extracellular matrix proteins, catenins, and p53 binding protein 1 are important for chemotherapy response in ovarian cancers. OMICS. 2009;13: 345–354.

71. Gadwal A, Purohit P, Khokhar M, Vishnoi JR, Pareek P, Choudhary R, et al. In silico analysis of differentially expressed-aberrantly methylated genes in breast cancer for prognostic and therapeutic targets. Clin Exp Med. 2023;23: 3847–3866.

72. Shen K, Ke S, Chen B, Zhang T, Wang H, Lv J, et al. Identification and validation of

biomarkers for epithelial-mesenchymal transition-related cells to estimate the prognosis and immune microenvironment in primary gastric cancer by the integrated analysis of single-cell and bulk RNA sequencing data. Math Biosci Eng. 2023;20: 13798–13823.

73. Hall RE, Horsfall DJ, Stahl J, Vivekanandan S, Ricciardelli C, Stapleton AMF, et al. Apolipoprotein-D: a novel cellular marker for HGPIN and prostate cancer. Prostate. 2004;58: 103–108.

74. Lim H, Kim SI, Kim EN, Lee M, Lee C, Kim J-W, et al. Tissue Expression and Prognostic Role of CXCL12 and CXCR4 in High-grade Serous Ovarian Carcinoma. Anticancer Res. 2023;43: 3331–3340.

75. Wei L, Ye H, Li G, Lu Y, Zhou Q, Zheng S, et al. Cancer-associated fibroblasts promote progression and gemcitabine resistance via the SDF-1/SATB-1 pathway in pancreatic cancer. Cell Death Dis. 2018;9: 1065.
